# *Mycobacterium tuberculosis* DosS binds H_2_S through its Fe^3+^ heme iron to regulate the DosR dormancy regulon

**DOI:** 10.1016/j.redox.2022.102316

**Published:** 2022-04-20

**Authors:** Ritesh R. Sevalkar, Joel N. Glasgow, Martín Pettinati, Marcelo A. Marti, Vineel P. Reddy, Swati Basu, Elmira Alipour, Daniel B. Kim-Shapiro, Dario A. Estrin, Jack R. Lancaster, Adrie J.C. Steyn

**Affiliations:** a Department of Microbiology, University of Alabama at Birmingham, Birmingham, AL, USA; b Centers for AIDS Research and Free Radical Biology, University of Alabama at Birmingham, Birmingham, AL, USA; c Universidad de Buenos Aires, Facultad de Ciencias Exactas y Naturales, Departamento de Química Inorgánica, Analítica y Química Física, Buenos Aires, Argentina; d CONICET-Universidad de Buenos Aires, Instituto de Química Física de los Materiales, Medio Ambiente y Energía (INQUIMAE), Buenos Aires, Argentina; e Universidad de Buenos Aires, Facultad de Ciencias Exactas y Naturales, Departamento de Química Biológica, Buenos Aires, Argentina; f CONICET-Universidad de Buenos Aires, Instituto de Química Biológica (IQUIBICEN), Buenos Aires, Argentina; g Department of Physics, Wake Forest University, Winston-Salem, NC, USA; h Department of Pharmacology & Chemical Biology, Vascular Medicine Institute, University of Pittsburgh School of Medicine, Pittsburgh, PA, USA; i Africa Health Research Institute, University of KwaZulu-Natal, Durban, South Africa

**Keywords:** Hydrogen sulfide, Tuberculosis, Dormancy regulon, Two-component system, DosS

## Abstract

*Mycobacterium tuberculosis* (*Mtb*) senses and responds to host-derived gasotransmitters NO and CO via heme-containing sensor kinases DosS and DosT and the response regulator DosR. Hydrogen sulfide (H_2_S) is an important signaling molecule in mammals, but its role in *Mtb* physiology is unclear. We have previously shown that exogenous H_2_S can modulate expression of genes in the Dos dormancy regulon via an unknown mechanism(s). Here, we test the hypothesis that *Mtb* senses and responds to H_2_S via the DosS/T/R system. Using UV–Vis and EPR spectroscopy, we show that H_2_S binds directly to the ferric (Fe^3+^) heme of DosS (K_D_^app^ = 5.30 μM) but not the ferrous (Fe^2+^) form. No interaction with DosT(Fe^2+^-O_2_) was detected. We found that the binding of sulfide can slowly reduce the DosS heme iron to the ferrous form. Steered Molecular Dynamics simulations show that H_2_S, and not the charged HS^−^ species, can enter the DosS heme pocket. We also show that H_2_S increases DosS autokinase activity and subsequent phosphorylation of DosR, and H_2_S-mediated increases in Dos regulon gene expression is lost in *Mtb* lacking DosS. Finally, we demonstrate that physiological levels of H_2_S in macrophages can induce DosR regulon genes via DosS. Overall, these data reveal a novel mechanism whereby *Mtb* senses and responds to a third host gasotransmitter, H_2_S, via DosS(Fe^3+^). These findings highlight the remarkable plasticity of DosS and establish a new paradigm for how bacteria can sense multiple gasotransmitters through a single heme sensor kinase.

## Graphical abstract

**Figure:**
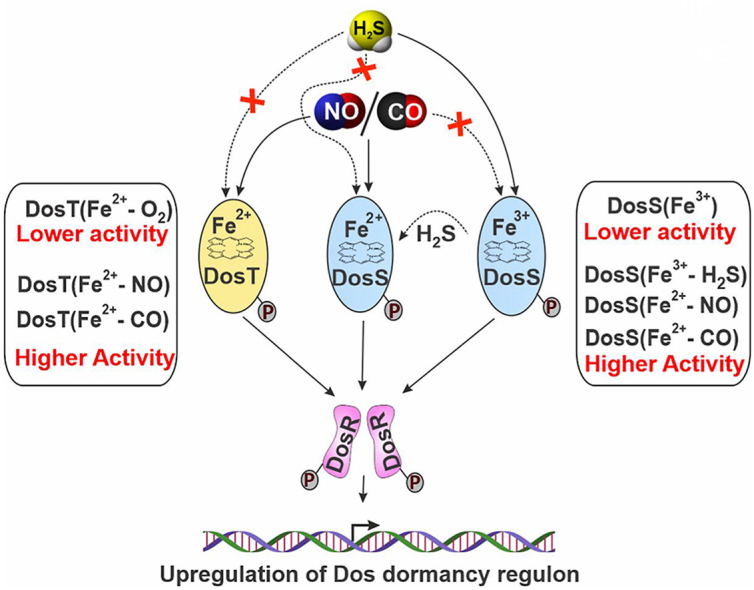

## 1 Introduction

Tuberculosis (TB) is a global epidemic responsible for ∼1.4 million deaths annually [1]. *Mycobacterium tuberculosis* (*Mtb*)*,* the causal agent of TB, can persist in a state of clinical latency for decades. *Mtb* survives and establishes an infection due, in part, to its ability to sense and respond to host defenses in the lung, including host-generated gasotransmitters. Carbon monoxide (CO) and nitric oxide (NO) are critical components of the host defense to clear the pathogen and are important to the outcome of *Mtb* infection [2,3]. The most recent addition to the list of gasotransmitters is hydrogen sulfide (H_2_S). Notably, enzymes required for the generation of NO, (nitric oxide synthase [iNOS]) [3], CO (heme oxygenase-1 [HO-1]) [2,4,5], and H_2_S (cystathionine β-synthase [CBS] [6], cystathionine γ-lyase [CSE] [6], and 3-mercaptopyruvate sulfur transferase [3-MPST]) [6], are upregulated in the lungs of *Mtb*-infected mice and human TB patients. The increased levels of these enzymes suggest an abundance of NO, CO, and H_2_S at the primary site of infection.

**Fig. 1:**
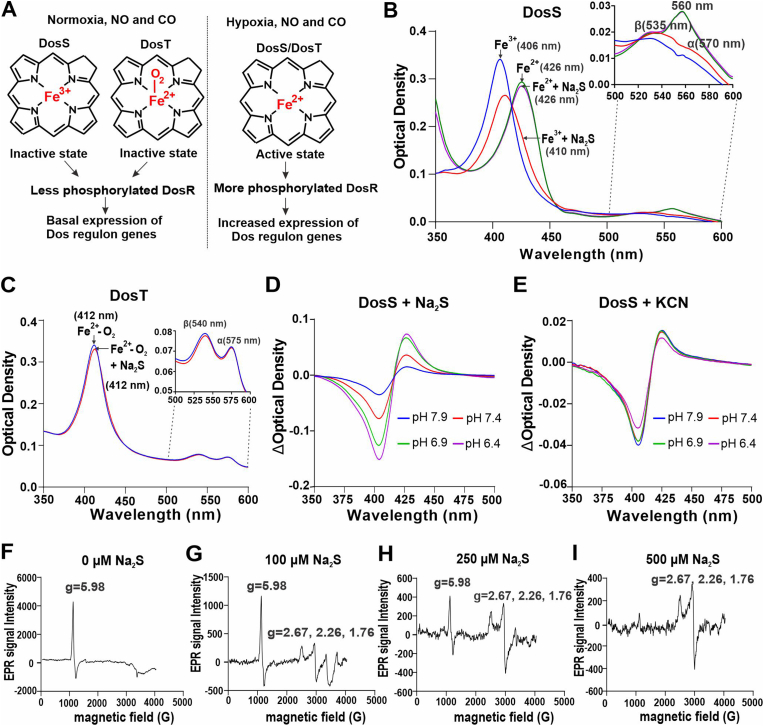
Characterization of DosS binding to H_2_S. (A) Depictions of recombinant DosS and DosT sensing of O_2_, NO and CO under various conditions. (B) Representative UV–Visible absorption spectra of recombinant DosS(Fe^3+^) alone (3 μM; *λ_max_* = 406 nm, blue curve) or in the presence of 100 μM Na_2_S (*λ_max_* = 410 nm, red curve), the DosS(Fe^2+^) in the presence of 400 μM DTH (green (*λ_max_* = 426 nm, curve) and the DosS(Fe^2+^) in the presence of 400 μM DTH and 100 μM Na_2_S (*λ_max_* = 426 nm, orange curve). (*Inset*) Absorption spectra replotted to highlight the α and β absorption peaks of DosS(Fe^3+^) (α at 570 nm and β at 535 nm), and DosS(Fe^2+^) (α at 560 nm). (C) Representative UV–Visible absorption spectra of recombinant DosT (3 μM) in the Fe^2+^-O_2_ form (*λ_max_* = 412 nm, blue curve) and the Fe^2+^-O_2_ form in the presence of 100 μM Na_2_S (*λ_max_* = 412 nm, red curve). (*Inset*) Absorption spectra replotted to highlight the α (575 nm) and β (540 nm) peaks. (D) Changes in the UV–Visible absorption spectra of DosS(Fe^3+^) (3 μM) resulting from the addition of 25 μM Na_2_S at different pH conditions, relative to DosS(Fe^3+^) alone. (E) Changes in the UV–Visible absorption spectra of DosS(Fe^3+^) (3 μM) resulting from the addition of 10 μM KCN at different pH conditions, relative to DosS alone. (F–I) EPR spectroscopic analysis of recombinant DosS showing a single axial peak characteristic of paramagnetic high-spin Fe^3+^ heme iron. DosS alone (F), and in the presence of increasing concentrations of Na_2_S (G–I) which shows the appearance of additional peaks characteristic of the low-spin state of heme iron. UV–Vis spectra are representative of at least 5 independent measurements. (For interpretation of the references to colour in this figure legend, the reader is referred to the Web version of this article.)

The role of NO and CO in TB pathogenesis is well studied compared to that of H_2_S. We have recently shown that *Mtb*-infected mice deficient in the H_2_S-producing enzyme CBS [7] or CSE [6] survive significantly longer with reduced organ burden, suggesting that host-generated H_2_S is beneficial for *Mtb in vivo*. Bacterial two-component regulatory systems sense changes in the host environment and mediate adaptive genetic responses. *Mtb* encodes the DosS/T/R system, which is comprised of heme-containing sensor kinases DosS and DosT and their cognate transcriptional response regulator DosR [8] that regulates the ∼48-member DosR dormancy regulon. DosS has only basal activity in the oxidized/met (Fe^3+^) form and has maximal activity in the ferrous (Fe^2+^) form which can bind CO or NO to keep DosS in the active state [9]. DosT is an oxygen sensor and is inactive in its oxy-bound form which is activated upon loss of O_2_ or direct binding of NO or CO to the heme iron in the reduced (Fe^2+^) state [9,10] (Fig. 1A).

We recently reported that exposure of *Mtb* cells to exogenous H_2_S induces the DosR dormancy regulon as well as genes that regulate cysteine metabolism and intracellular copper levels [7]. Furthermore, our recent report demonstrating that *Mtb* is capable of producing H_2_S [11] reveals additional complexity as it implicates endogenous and host-derived H_2_S, in addition to host-derived NO, CO and O_2_, in regulation of the DosR dormancy regulon. While H_2_S can chemically modify biomolecules directly [[12], [13], [14]], we considered the possibility that alterations in gene expression in response to H_2_S are mediated by regulatory proteins in *Mtb*. Since H_2_S is known to bind the iron in heme-containing proteins [[15], [16], [17], [18], [19]], and because DosS was among the Dos regulon genes induced upon exposure to H_2_S [7], we hypothesize that DosS and/or DosT sense and respond to H_2_S to induce the DosR dormancy regulon. To examine the interaction of H_2_S with DosS and DosT, we used ultraviolet–visible (UV-Vis) and electron paramagnetic resonance (EPR) spectroscopy. We also examined how H_2_S modulates DosS autokinase and phosphate transfer, and *Mtb* gene expression *in vitro*. Lastly, we determined whether *Mtb* senses H_2_S during macrophage infection. We expect that the findings in this work will lead to an improved understanding of *Mtb* persistence.

## 2 Results

### 2.1 DosS in the Fe^3+^ form binds H_2_S

DosS and DosT contain heme and exhibit specific absorption characteristics in the UV–Vis range, which are altered upon interaction between the heme iron and ligands like NO and CO [9,20]. To determine whether *Mtb* DosS or DosT sense H_2_S via its heme iron, we monitored spectral changes of the ferric (Fe^3+^) and ferrous (Fe^2+^) forms of recombinant DosS and DosT in the presence of sulfide (here, we define sulfide as H_2_S and HS^−^) following addition of sodium sulfide (Na_2_S). Addition of sulfide red shifts the Soret peak of the Fe^3+^ form of DosS from 406 nm to 410 nm, indicative of a high-spin to low-spin transition [21], with increased peak intensities of the α (570 nm) and β (535 nm) bands (Fig. 1B), similar to spectral changes observed upon sulfide binding in other heme-containing proteins (Table S1). Reduction of DosS to the Fe^2+^ form using sodium dithionite (DTH) shifts the Soret peak to 426 nm with emergence of a new peak at 560 nm, as observed previously [9,22]. However, the absorption pattern of DTH-reduced DosS remains unchanged in the presence of sulfide (Fig. 1B). Similarly, addition of sulfide does not alter the absorption spectrum of DosT, where the heme iron remains in the oxy-bound state (Fe^2+^-O_2_) (Fig. 1C) [9,22]. These data suggest that sulfide directly interacts with the Fe^3+^ form of DosS. This is mechanistically distinct from the binding of NO and CO, which bind the heme iron of DosS and DosT in the Fe^2+^ state.

H_2_S is in protonation equilibrium with HS^−^ in solution with a pKa value of 7.01 [23]. To determine whether H_2_S or anionic hydrosulfide (HS^−^) binds to DosS(Fe^3+^), we monitored the UV–Vis spectra of DosS at pH values above and below the pKa of H_2_S (pH 7.9–6.4) in the presence of 25 μM Na_2_S. Notably, as the pH decreased we observed lower peak intensities in the 405 nm range, indicating reduced levels of unbound DosS, and increases in the ∼420 nm range corresponding to sulfide-bound DosS (Fig. 1D). To confirm that increased binding of H_2_S below its pKa results from increased concentrations of H_2_S via protonation of HS^−^, and not from pH effects on DosS protein, we examined the effects of pH on the binding of another low spin ferriheme ligand, cyanide (CN^−^), whose pKa is outside the neutral range (pKa = 9.2). As shown in Fig. 1E, the spectra of DosS in the presence of 10 μM CN^−^ shows that CN^−^ binding is virtually unchanged in the pH range of 7.9–6.4, in contrast to marked changes upon sulfide binding (Fig. 1D) under identical conditions. This indicates that DosS is negligibly influenced by pH changes in this range and confirms that H_2_S, and not HS^−^, is the sulfide species that initially binds to the heme iron of DosS.

Iron is paramagnetic in its Fe^3+^ state. Ligand binding to the heme iron results in perturbations in the *d*-orbitals that can be monitored by EPR spectroscopy [9,24]. To confirm that H_2_S binds directly to the Fe^3+^ form of DosS, we compared the EPR spectra of DosS before and after exposure to sulfide. DosS alone gives a strong axial feature centered at g = 5.98, which is indicative of Fe^3+^ in the high-spin (S = 5/2) state (Fig. 1F) [9,25] (Table S1). The addition of increasing concentrations of sulfide results in the conversion of the g = 5.98 high-spin signal into a rhombic low-spin signal with g values of 2.67, 2.26, and 1.76 (Fig. 1G–I), which are similar to low-spin sulfide-bound species reported for other heme-containing proteins (Table S1). Taken together, these results indicate that H_2_S is a ligand of the Fe^3+^ form of DosS and binding of H_2_S converts the Fe^3+^ heme iron from the high-spin state to low-spin state.

### 2.2 Molecular dynamics simulations show that H_2_S, but not HS^−^, enters the DosS heme pocket

**Fig. 2:**
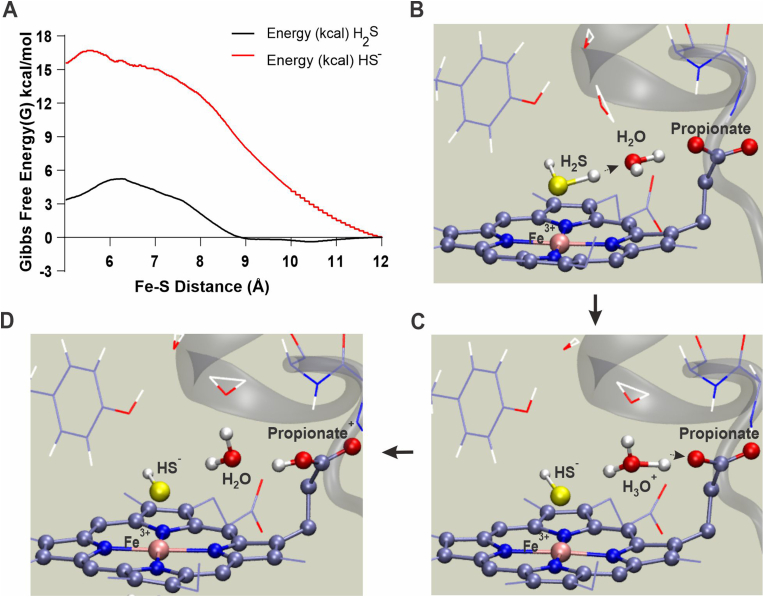
Molecular modeling of DosS interaction with H_2_S. (A) Calculated average association free energy profiles for H_2_S and HS^−^ as a function of intermolecular distance between the DosS heme iron and sulfur. Free energy values were generated by employing 98 separate trajectories for H_2_S and 89 trajectories for HS^−^ using a steered Molecular Dynamics (sMD) approach. (B–D) QM/MM MD simulation snapshots depicting the steps of a predicted proton transfer from H_2_S to a nearby heme propionate group through a water bridge within 0.3 ps of MD. The QM subsystem atoms are shown in ball and stick representation.

The DosS heme group is buried within a hydrophobic pocket that is enclosed within the N-terminal GAF-A domain [22,26]. Access to the hydrophobic heme pocket is limited, and ligand entry is influenced by the adjacent amino acid side chains [22]. Steered Molecular Dynamics (sMD) has been used to estimate association free energy required for H_2_S and HS^−^ to access the heme iron in *L. Pectinata* met-hemoglobin [27] and met-myoglobin [27,28]. Similarly, our sMD simulations estimate the free energy barriers for access to the DosS heme iron to be approximately 5.6 kcal/mol for H_2_S and 16.7 kcal/mol for HS^−^ (Fig. 2A). The much higher free energy barrier for HS^−^ indicates that the uncharged H_2_S species is strongly favored to enter the heme pocket.

Our sMD simulations predict that H_2_S accesses the heme iron by passing between Phe^98^ and Leu^114^ of the heme binding pocket (Fig. S1). These residues form a “gate” which when open allows H_2_S and a few water molecules into and out of the heme pocket. This is possible due to the neutral charge of H_2_S (Fig. S1 A-C, Movie S1). In contrast, our sMD simulations indicate that HS^−^ carries a strongly-bound solvation sphere that pushes the Phe^98^ and Leu^114^ side chains away, resulting in a considerable number of water molecules entering the heme pocket and increasing solvation of the heme active site (Fig. S1 D-F, Movie S2). The process of HS^−^ entry is energetically unfavorable, as indicated by a much higher predicted free energy barrier compared to H_2_S. Notably, this H_2_S entry “gate” is located away from the “water channel” identified by Cho, et al., in the DosS GAF-A domain structure [22]. Overall, our sMD modeling indicating more favorable heme access for H_2_S supports our UV–Vis data demonstrating increased binding of sulfide at lower pH (Fig. 1D).

Supplementary data related to this article can be found at https://doi.org/10.1016/j.redox.2022.102316.

The following are the supplementary data related to this article:

Multimedia component 1 Multimedia component 1

Multimedia component 2 Multimedia component 2

### 2.3 Quantum mechanics simulations show that H_2_S deprotonates following heme iron binding

Modeling studies of heme-containing proteins predict that H_2_S can deprotonate following binding to heme iron [27,28]. To characterize the heme-bound state of H_2_S in DosS, we employed combined quantum mechanics/molecular mechanics simulations with density functional theory calculations (QM[DFT]/MM). Table S2 shows the structural and electronic parameters of both H_2_S and HS^−^ bound states of DosS. As expected, the ligand-bound states display a low-spin ground state. The structural analysis shows that HS^−^ forms a tighter bond (a significantly smaller Fe–S distance, [d Fe–S = 2.17 Å]) due to a significant charge transfer (sigma donation). Further, the proximal Fe-His bond shows a slightly positive trans effect, displaying a slightly smaller distance compared to penta-coordinated heme (ca 2.12 Å) [29,30]. Interestingly, in the H_2_S-bound state, the two protons become asymmetric and a weakening of the S–H bond is observed (d Fe–S = 2.35 Å). On this basis, we evaluated the possibility of deprotonation of Fe-bound H_2_S using hybrid QM/MM simulations. Strikingly, a simulation duration of 1 ps (ps) was sufficient to observe deprotonation of Fe-bound H_2_S (Fig. 2B–D and Movie S3). The proton acceptor is a water molecule located opposite from the distal Tyr (i.e., close to the solvent-exposed heme edge) which subsequently transfers a proton to the heme propionate (Fig. 2D). The heme propionate is accessible to the solvent and can again be deprotonated or remain in a protonated state. Overall, these data strongly suggest that HS^-^ is the tighter binding and predominant bound sulfur species.

Supplementary data related to this article can be found at https://doi.org/10.1016/j.redox.2022.102316.

The following is the supplementary data related to this article:

Multimedia component 3 Multimedia component 3

### 2.4 H_2_S binds with low micromolar affinity and slowly reduces the DosS heme iron

**Fig. 3:**
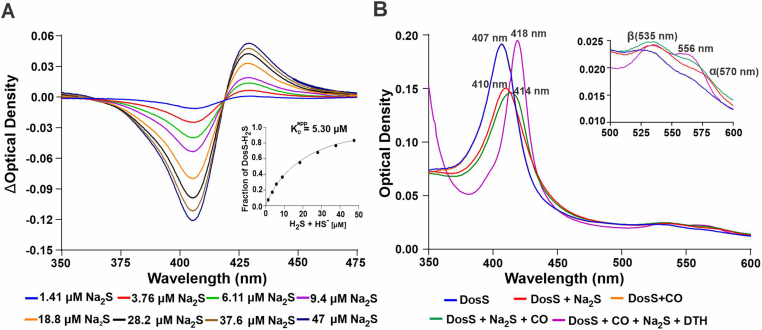
H_2_S binds with low micromolar affinity and slowly reduces the DosS heme iron. (A) Representative changes to the UV–Visible spectra of DosS(Fe^3+^) (3 μM) resulting from the addition of different concentrations of Na_2_S, relative to DosS without Na_2_S. Inset shows a substrate saturation curve of H_2_S binding to DosS(Fe^3+^) generated using UV–Vis absorption data points obtained from the titration DosS with increasing concentrations of Na_2_S. UV–Vis spectra are representative of at least 3 independent assays. (B) Representative UV–Visible spectra of recombinant DosS(Fe^3+^) (3 μM) (*λ_max_* = 407 nm, blue curve) 90 min after the addition of 30 μM CO (*λ_max_* = 407 nm, orange curve), 30 μM Na_2_S (*λ_max_* = 410 nm, red curve), CO + Na_2_S (*λ_max_* = 414 nm, green curve), or Na_2_S + CO+0.5 mM DTH (*λ_max_* = 418 nm, magenta curve). Inset shows absorption spectra replotted to highlight the α (570 nm) and β (535 nm) peaks. (For interpretation of the references to colour in this figure legend, the reader is referred to the Web version of this article.)

To determine the affinity of DosS for H_2_S, we next monitored changes in the absorbance spectrum of DosS in the Fe^3+^ state over a wide range of Na_2_S concentrations (Fig. 3A). Using the maximum change in absorbance at ∼408 nm, we generated a substrate saturation curve from which a K_D_^app^ value of 5.30 ± 0.35 μM was calculated for H_2_S binding to DosS (Fig. 3A and Inset). Notably, this K_D_^app^ value is similar to the K_D_^app^ value of 7.0 ± 0.4 μM reported for the H_2_S-met hemoglobin complex [18].

H_2_S is known to reduce heme iron to the Fe^2+^ state in proteins like myoglobin and hemoglobin [16]. Since DosS(Fe^2+^) is understood to be the active form of the kinase [9,20], it is important to determine whether H_2_S binding can reduce the DosS heme iron. In this regard, slow oxygen consumption has been observed following sulfide binding to ferrihemoglobin [31] or ferrimyoglobin [32], indicative of redox/oxygenation reactions with production of oxidized sulfur species such as persulfide, polysulfide and/or oxysulfur. Proposed mechanisms [31,33] include heme iron reduction to form ferroheme which in the presence of O_2_ will be readily autoxidized to the ferric form and rebind sulfide. Thus, the reduction of the DosS heme iron in the presence of oxygen may be undetectable. Indeed, we could not detect the formation of DosS(Fe^2+^) in the presence of 30 μM Na_2_S over 90 min (Fig. S2). We then used CO as “trap” to detect DosS(Fe^2+^) by forming the stable carbonmonoxy species [16,17]. As shown in Fig. 3B, addition of Na_2_S alone resulted in the expected shift in the Soret peak from 407 nm to 410 with increased peak intensity of the α (570 nm) and β (535 nm) bands, indicative of sulfide binding to Fe^3+^. As expected, no binding was observed in the presence of 30 μM CO, since CO binds only to DosS(Fe^2+^) [9]. Notably, addition of H_2_S and CO shifted to Soret peak from 407 nm to 414 nm, indicating the presence of ferrisulfide (*λ_max_* = 410 nm) with an approximately equal accumulation of carbonmonoxy iron (*λ_max_* = 418 nm). These data show that H_2_S can reduce the DosS heme iron during relatively long (t_1/2_ ≥ 90 min) incubation under aerobic conditions.

### 2.5 H_2_S increases DosS autokinase activity and DosR phosphorylation

**Fig. 4:**
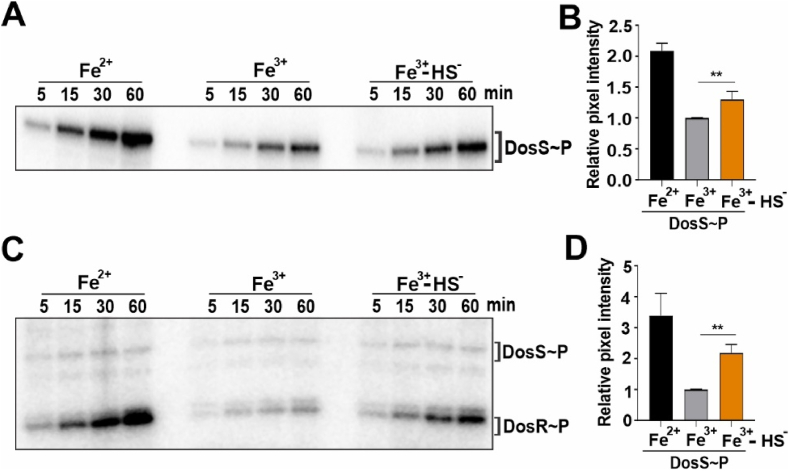
H_2_S stimulates DosS and DosR phosphorylation. (A) Representative autoradiogram of PAGE-resolved recombinant DosS following autophosphorylation in the presence of γ-^32^P-ATP alone (left and center lanes) or with γ-^32^P-ATP in the presence of 100 μM Na_2_S (right lanes). (B) Densitometric quantitation of DosS bands (n = 3) at 60 min. (C) Representative autoradiogram of PAGE-resolved recombinant DosS and DosR following phosphorylation of DosR by γ-^32^P-labeled DosS alone (left and center lanes) or with γ-^32^P-labeled DosS in the presence of 100 μM Na_2_S (right lanes). These reactions were performed by adding γ-^32^P-labeled ATP to a reaction containing both DosS and DosR and analyzed at different time points. (D) Densitometric quantitation of DosR bands (n = 3) at 60 min. Data in (B) and (D) are shown as the mean ± SEM and were analyzed using one-way ANOVA with Tukey's multiple comparisons test performed using GraphPad Prism version 9. **p < 0.01. Autoradiograms are representative of at least 3 independent assays.

To address the question of whether H_2_S binding can alter DosS kinase activity, we performed kinase assays using γ-^32^P-labeled ATP and recombinant DosS. We determined relative autokinase activities of DosS (Fe^2+^), DosS (Fe^3+^), and DosS (Fe^3+^-HS^-^) at 5–60 min following addition of γ-^32^P labeled ATP. Compared to the Fe^3+^ form of DosS, which is considered the least active form [9], the Fe^3+^-HS^-^ form of DosS exhibited increased autokinase activity. In these assays, the Fe^2+^ form of DosS showed the highest autokinase activity (Fig. 4A). Densitometric analysis of autokinase radiograms shows that the activity of the Fe^3+^-HS^-^ form of DosS is increased by ∼30% at all time points compared to unbound DosS in the (Fe^3+^) form (Fig. 4B). Similarly, we determined the autokinase activity of DosT-Fe^2+^, and DosT-Fe^2+^-O_2_ with or without addition of 100 μM Na_2_S. We observed no increase in DosT autokinase activity following addition of Na_2_S (Fig. S3).

Next, we sought to determine whether the H_2_S-mediated increase in DosS autokinase activity results in augmented phosphorylation of DosR, the cognate response regular of DosS. First, to standardize our transphosphorylation assays, unphosphorylated recombinant DosR was added to reaction buffer containing γ-^32^P-labeled DosS. Surprisingly, the phosphorylation of DosR by labeled DosS was extremely rapid, and was completed within seconds (Fig. S4). Therefore, to observe differences in the rates of phosphate transfer of DosS(Fe^2+^), DosS(Fe^3+^), and DosS(Fe^3+^-HS^-^), unphosphorylated DosS and DosR were added to the reaction buffer prior to the addition of γ-^32^P-labeled ATP. Under these conditions, we observed increased phosphorylation of DosR in the presence of DosS (Fe^3+^-HS^-^) compared to DosR phosphorylation in the presence of DosS (Fe^3+^) at all time points measured. Maximum phosphorylation of DosR was observed in the presence of DosS(Fe^2+^) (Fig. 4C). Densitometric analysis of transphosphorylation radiograms shows an ∼2-fold increase in labeled DosR in the presence of DosS(Fe^3+^-HS^-^) compared to the Fe^3+^ form of DosS (Fig. 4D). Overall, these data indicate that the binding of sulfide to DosS increases its autokinase activity. Given the extremely rapid transfer of phosphate from DosS to DosR, we conclude that increased DosR phosphorylation is attributable primarily to changes in DosS autokinase activity.

**Fig. 5:**
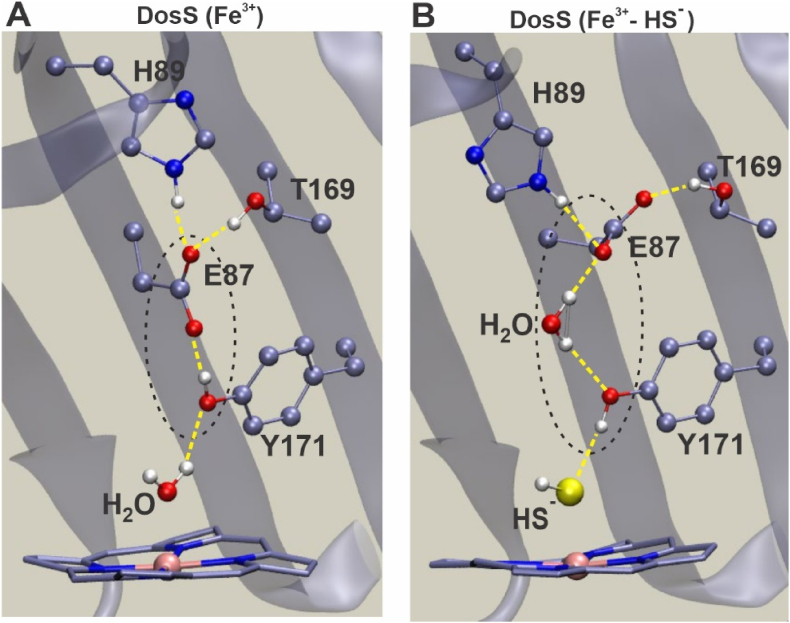
Sulfide binding alters the hydrogen bonding network in the DosS heme pocket (A) MD-modeled hydrogen bonding network in the distal domain of ferric (Fe^3+^) DosS showing an intact H-bond between glutamate (E87) and tyrosine (Y171). (B) MD-modeled hydrogen bonding network in the distal domain of ferric (Fe^3+^) DosS in the presence of sulfide showing disrupted H-bonding between glutamate (E87) and tyrosine (Y171). Predicted changes in the H-bonding patterns may lead to structural changes which alter DosS kinase activity upon sulfide binding.

Our findings that sulfide binding increases autokinase activity and that HS^−^ is likely the predominant bound ligand have implications for the structural basis of kinase domain activation. To elucidate a mechanism by which sulfide binding increases DosS autokinase activity, we employed MD modeling to detect structural differences between DosS(Fe^3+^) and DosS(Fe^3+^-HS^-^). Our analysis indicates that the hydrogen bonding network distal to the heme is significantly different between the ferric high spin (off-state) and the Fe^3+^-HS^-^ low spin state (Fig. 5), but not the Fe^3+^-H_2_S state. In the Fe^3+^ state of DosS, there is a loosely coordinated water molecule which cannot act as a hydrogen bond acceptor for Tyr^171^. Therefore, the hydroxyl group of Tyr^171^ rotates upward and instead forms a tight hydrogen bond with Glu^87^ (Fig. 5A). In contrast, when HS^−^ is bound to DosS in the Fe^3+^ state, Tyr^171^ hydroxyl group rotates downward and establishes a tight hydrogen bond with HS^−^ with the negative sulfur atom as the hydrogen bond acceptor. This results in the release of Glu^87^, which then moves closer to, and establishes a tight hydrogen bond with, His^89^ and Thr^169^ resulting in changes in their relative position, particularly for the loop in the peptide backbone in which H^89^ is located. Disruption of the hydrogen bond between Tyr^171^ and Glu^87^ is further promoted by the presence of water molecules between them (Fig. 5B). These observations suggest that the DosS off-state is characterized by strong Tyr^171^-Glu^87^ interaction, while the on-state is characterized by a strong Tyr^171^-HS^-^ interaction that releases Glu^87^, which is consistent with the observation that CO/NO-bound DosS is active and shows the disruption of the hydrogen bonding network between Tyr^171^-Glu^87^-His^89^ [26]. Overall it appears that signal transmission to the histidine kinase domain is initiated by disruption of Glu^87^-Tyr^171^ H-bonding and further amplified by positional changes in the His^89^-containing loop.

### 2.6 DosS senses H_2_S to regulate the Dos dormancy regulon

**Fig. 6:**
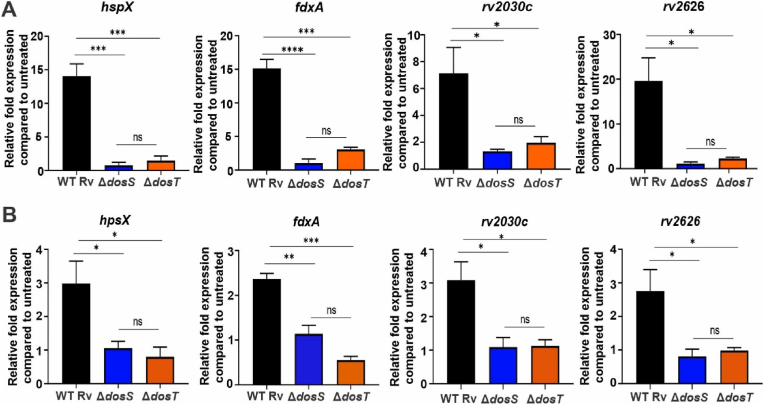
Effect of H_2_S on expression of DosR regulon genes. (A) Representative gene expression analysis of selected DosR regulon genes in WT, ΔdosS and ΔdosT *Mtb* cells exposed to 50 μM Na_2_S for 30 min, relative to unexposed *Mtb* cells (n = 3). (B) Expression of selected DosR regulon genes in WT, ΔdosS, and ΔdosT *Mtb* isolated from infected RAW 264.7 macrophages grown in cysteine/methionine-free medium containing 400 μM GYY4137 for 24 h, relative to *Mtb* isolated from unexposed macrophages (n = 3). Data are shown as the mean ± SEM and were analyzed using one-way ANOVA with Tukey post-hoc test using GraphPad Prism version 9. *p < 0.05, **p < 0.01, ***p < 0.001, and ****p < 0.0001, ns = p ≥ 0.05.

**Fig. 7:**
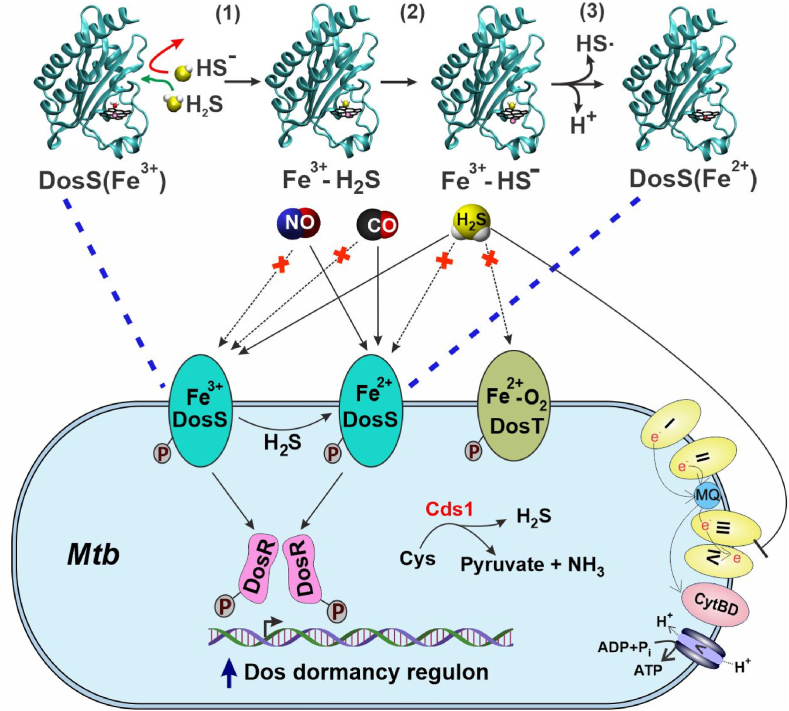
Schematic of a proposed mechanism of H_2_S sensing by *Mtb.* H_2_S derived from the host or by *Mtb* via Cysteine desulfhydrase 1 (Cds1, Rv3684) can enter the DosS heme pocket (green arrow). (1) Based on sMD modeling, the charged HS^−^ species does not enter the heme pocket (red arrow). Upon entering the heme pocket, H_2_S binds to the heme iron of DosS(Fe^3+^) only, demonstrating that sulfide sensing is different than for NO and CO, which bind only DosS(Fe^2+^). In contrast, DosT does not bind H_2_S. (2) QM/MM MD simulations suggest that H_2_S deprotonates to form bound hydrosulfide anion (HS^−^). The proton acceptor is predicted to be a water molecule located opposite from the distal Tyr (i.e., close to the solvent-exposed heme edge) which then subsequently transfers a proton to the heme propionate. (3) The heme propionate donates a H_2_S-derived proton to the solvent and sulfide can slowly reduce the DosS heme iron, followed by the release of a hydrosulfide radical (HS^**.**^) to the solvent. DosS(Fe^2+^) can then bind CO or NO. DosS autokinase activity is lowest for unbound DosS(Fe^3+^) and is increased via binding of H_2_S to DosS(Fe^3+^) or reduction to DosS(Fe^2+^), which could ultimately lead to increased expression of DosR dormancy regulon genes. At higher concentrations, H_2_S inhibits Complex IV, resulting in rerouting of electrons through cytochrome *bd* oxidase (CytBD). (For interpretation of the references to colour in this figure legend, the reader is referred to the Web version of this article.)

To determine whether the H_2_S-mediated activation of DosS is sufficient to increase expression of DosR regulon genes, we exposed WT *Mtb*, *Mtb* Δ*dosS,* and *Mtb* Δ*dosT* (*dosS* and *dosT* deletion mutants, respectively [34]) to 50 μM Na_2_S. This corresponds to an H_2_S concentration of ∼15.8 μM the culture media, based on calculations accounting for headspace volume and Henry's Law constant for H_2_S (see Materials and Methods). This was followed by quantitation of mRNA transcripts of representative DosR regulon genes. As shown in Fig. 6A, *hspX*, *fdxA*, *rv2030c* and *rv2626* transcript levels were markedly increased in WT *Mtb* while transcript levels in *Mtb* Δ*dosS* were unchanged by Na_2_S exposure. Transcript levels in *Mtb* Δ*dosT* cells were also reduced compared to WT, but trended higher than those in Δ*dosS.* Of note, we previously observed a similar pattern of reduced induction of DosR regulon genes in Δ*dosS* and Δ*dosT* cells following exposure to GYY4137 [7]. These data suggest that DosT is required for H_2_S-dependent, DosS-mediated transcriptional responses in *Mtb.* This is reminiscent of studies that show Δ*dosS* and Δ*dosT* cells have different capacities for sensing NO [5] or CO [4,5] even though both sensor kinases bind NO and CO, suggesting that other factors are involved in modulating sensing (see Fig. 7).

H_2_S is produced enzymatically by *Mtb* [11] and the host [35,36] using Cys as a substrate. We used the fluorescent WSP-5 H_2_S-sensing probe [37] to monitor relative H_2_S levels in RAW 264.7 macrophage cells grown in media containing 0–2 mM L-Cys. Intracellular fluorescence was increased in RAW 264.7 macrophages grown in L-Cys-containing media compared to cysteine-free media, with maximum signal observed at 200 μM L-Cys (Fig. S5), which is within the physiological range of 30–260 μM [[38], [39], [40]].

Next, to determine if *Mtb* can respond to physiological levels of endogenous H_2_S, RAW 264.7 macrophages were grown in cysteine-free media or media containing 200 μM L-Cys and infected with WT or Δ*dosS Mtb*. At 24 h post-infection, intracellular bacilli were recovered, and RNA was extracted. As shown in Fig. S6, we observed marked increases in *hspX*, *fdxA*, *rv2030c,* and *rv2626* transcripts in WT *Mtb*, but not Δ*dosS Mtb.*

To confirm that *Mtb* in infected macrophages can sense H_2_S via DosS to mediate induction of DosR regulon genes, RAW 264.7 macrophages were infected with WT *Mtb*, *Mtb* Δ*dosS* or *Mtb* Δ*dosT* and exposed to the slow H_2_S donor GYY4137. Transcript levels of DosR regulon genes were increased in WT *Mtb* isolated from GYY4137-exposed macrophages, but not in isolated *Mtb* Δ*dosS* or Δ*dosT* (Fig. 6B). Taken together, these results are consistent with H_2_S-dependent increases in expression of DosR genes via direct binding to DosS in which DosT plays an indirect role.

## 3 Discussion

The *Mtb* DosS/T/R signal transduction system senses host-derived dormancy signals, NO [9,10,34], O_2_ [34,41], and CO [4,5] to induce the 48-gene DosR dormancy regulon. Here, we report that *Mtb* can sense a third gasotransmitter, H_2_S, to induce the Dos dormancy regulon. Importantly, we show that DosS is capable of sensing H_2_S at levels produced by macrophages, resulting in the upregulation of key dormancy regulon genes. We also show that *Mtb* DosS, but not DosT, senses H_2_S directly via its heme iron in the ferric state to modulate its autokinase activity resulting in increased phosphorylation of DosR. The ability of sulfide to bind DosS ferric heme iron is clearly distinct from that of NO and CO, which bind DosS only when its heme iron is reduced and represents a fundamentally important functional difference between H_2_S, and NO, CO and O_2_ sensing by DosS. Thus, DosS exhibits remarkable plasticity in sensing multiple gasotransmitters regardless of the oxidation state of the heme iron to ensure induction of the DosR dormancy regulon during infection. These findings establish a new paradigm for how bacteria sense multiple signaling molecules with distinct physicochemical properties through a single heme sensor kinase.

The importance of H_2_S as a signaling molecule in the regulation of numerous physiological functions in mammals, including immunity, is well established [42,43]. Hence, it is reasonable that bacterial pathogens have evolved the capacity to detect H_2_S to reprogram their transcriptomes to subvert the immune response. However, while numerous signaling studies have been performed in bacteria including, but not limited to, *E. coli,* a gut organism continuously exposed to H_2_S, little is known regarding whether H_2_S has a direct signal transduction function in bacteria. More specifically, the lack of evidence supporting the ability of bacteria to sense and respond to H_2_S via heme sensor kinases in two-component systems [44,45] represents a major gap in our knowledge.

Of note, H_2_S is produced at the site of *Mtb* infection, as demonstrated by the presence of H_2_S-producing enzymes CSE and 3-MPST around cavities and necrotic lesions in the lungs of TB patients [6]. Since H_2_S is highly diffusible, intracellular and extracellular *Mtb* will be exposed to host-generated H_2_S. *Mtb* is also exposed to NO [3], CO [2] and hypoxia [46] in the lungs of TB patients depending on disease stage. NO, CO and H_2_S are produced at different levels and times throughout the course of infection, which likely vary depending on the particular microanatomical location and pathology induced by *Mtb*. This, coupled with differing on- and off-rates of these molecules for DosS and DosT heme iron, strongly suggest that the *Mtb* DosR dormancy regulon is induced throughout the course of infection. Studies in the macaque model of inhalation TB have shown that *Mtb* Δ*dosS,* Δ*dosR* and Δ*dosT* mutants are attenuated [47]. However, a *Mtb* Δ*dosS* mutant, but not Δ*dosR* or Δ*dosT* mutants, was shown to be attenuated in C3HeB/FeJ mice and macrophages [48]. Notably, these authors demonstrated that DosS phosphorylates proteins other than DosR, suggesting that DosS may modulate expression of genes outside the DosR dormancy regulon [49]. Intriguingly, we have shown that exposure of *Mtb* cells to exogenous H_2_S induces the copper regulon [7], which is part of the enduring hypoxic response (EHR) [50] that is independently regulated and induced 4–7 days after the DosR dormancy regulon [50]. Induction of *Mtb* copper efflux pumps by H_2_S is not surprising since copper has a high affinity for H_2_S, which generates copper sulfide species. Importantly, our findings suggest a role for exogenous H_2_S in establishment of the EHR. Furthermore, our recent finding that *Mtb* produces H_2_S [11] provides additional insight into the complexity of regulation of the DosR regulon. For example, since L-Cys is a substrate for H_2_S production, it suggests that in addition to CBS-, CSE- and 3-MPST-generated H_2_S, the availability of *in vivo* sulfur substrates such as L-Cys will also modulate the DosR regulon. Lastly, since H_2_S interacts with ferric DosS to induce the DosR regulon, it suggests that induction of the DosR regulon *in vivo* can occur even in the presence of O_2_.

Several lines of evidence provide insight into the mechanism whereby *Mtb* senses H_2_S. Firstly, we found that DosS, but not DosT, is a H_2_S sensor as the latter is in the ferrous oxy-bound state (Fe^2+^-O_2_) under normoxic conditions. Secondly, sulfide binds DosS(Fe^3+^) under normoxic conditions as demonstrated by UV–Vis and EPR spectroscopy. These data also show that direct H_2_S binding to the ferric iron changes its spin state from high to low. Further, sulfide can slowly reduce the heme iron with consequent sulfide oxidation. Our findings are consistent with several reports showing that sulfide reacts with heme iron in the ferric (Fe^3+^) state [[15], [16], [17], [18], [19]]. This may allow induction of the DosR regulon at earlier stages of infection prior to the onset of hypoxia when ferrous DosS can respond to NO and CO only. Thirdly, our MD modeling using the DosS GAF-A domain X-ray structure (PDB code 2W3E) [22] provides key insight into the molecular interactions of sulfide with DosS. Consistent with our sMD modeling of other hemoproteins [27,28] H_2_S has more favorable access to the DosS heme than does HS^−^, consistent with the contrasting effects of pH on the binding of sulfide (pKa = 7.01) reported here and elsewhere [17,31] which we compared to cyanide (pKa = 9.2). This selectivity is due to the strong hydration of HS^−^ compared to H_2_S which significantly hinders HS^−^ entry into the hydrophobic pocket, particularly *via* a “gate” comprised of Phe^98^ and Leu^114^ side chains. Further, QM[DFT]/MM calculations demonstrate a stronger Fe–S bond for HS^−^ than for H_2_S, suggesting deprotonation is induced upon binding of H_2_S. Indeed, our hybrid QM/MM simulations indicate proton transfer from H_2_S via a water molecule to a heme propionate resulting in bound HS^−^ as the final state. In DosS, proton acceptor groups in the distal position near the bound sulfide may enhance heme iron reduction, as has been suggested for other hemoproteins [51].

In hemoglobin [31] and myoglobin [32] rapid sulfide binding is followed by slower reduction of the heme iron and O_2_ consumption resulting from complex sulfur redox/oxygenation reactions which are not fully delineated [31,33]. By employing CO as a ferroheme trap [16,17] we demonstrate sulfide-induced heme reduction (assuming relatively rapid heme CO accessibility); however, the rate is slow, t_1/2_ ≥ 90 min (Fig. 3B). Although not rapid compared to binding, this reduction suggests an additional possible mechanism for sulfide-induced dormancy induction, as well as generation of persulfide and/or other sulfane signaling species [52] by catalytic action of DosS in the presence of O_2_.

To the best of our knowledge, this is the first report of a bacterial heme sensor kinase that binds H_2_S to increase its catalytic activity under physiologically relevant conditions. An important remaining question is how the binding of H_2_S to DosS increases autokinase activity. Given that a complete crystal structure for DosS has not been reported, a detailed study of the structural changes in the kinase domain upon H_2_S binding remains challenging. Nonetheless, structural studies probing the mechanism by which DosS activity is modulated by the redox state of, and ligand binding to, the heme iron have shown that an intact hydrogen bonding network comprised of Tyr^171^-Glu^87^-His^89^ is important for inhibition of DosS kinase activity, as seen in the Fe^3+^ state [22]. Disruption of this network upon reduction of the heme iron and subsequent binding of NO or CO result in DosS conformations that favor kinase activity [22,26,53,54]. Our MD- and QM/MM-based modeling of the DosS GAF-A domain predicts that HS^−^ ligation to DosS disrupts the distal Tyr^171^-Glu^87^-His^89^ hydrogen bonding network. This is consistent with previously published proposals regarding signal transmission between the heme and autokinase domains [26] and with our finding of H_2_S-stimulated autokinase and DosR phosphorylation.

Our finding that H_2_S binds DosS(Fe^3+^) only, and not DosT which is in the Fe^2+^-O_2_ state, demonstrates that the mechanism of H_2_S sensing is distinct from NO and CO sensing, since these molecules bind DosS(Fe^2+^) only. We [9] and others [22,55,56] have shown that under normoxia DosS exists in the Fe^3+^ state and requires a reductant to generate DosS(Fe^2+^) that binds NO or CO. Ferredoxin (FdxA) [58], reduced flavin nucleotides [22], and chorismate synthase (CS) have been posited as reductants of ferric DosS [22,57]. FdxA and CS may be candidate reductants since *fdxA* is part of the DosR regulon which is upregulated under hypoxic conditions and CS accelerates NADH-dependent FMN reduction. These potential reducing systems for DosS(Fe^3+^) may represent an additional aspect of DosR regulon induction, and suggests that in the absence of a reducing system, the Fe^3+^ form of DosS can still induce the DosR regulon via binding of H_2_S. Hence, in the presence of NO, CO or H_2_S, the DosR regulon will be induced via DosS regardless of whether the heme iron is in the Fe^2+^ or Fe^3+^ (unligated or ligated) state. Thus, these findings suggest that DosS signaling arising due a change in redox state and ligand binding are equally important. This may have important consequences *in vivo* and again suggests that the DosR dormancy regulon is induced through most of the course of infection. It has been shown that NO and hypoxia [10,59], and likely CO, inhibit respiration, and all three factors induce the DosR regulon [5,10,59]. However, we have shown that H_2_S increases *Mtb* respiration at low concentrations [7] and induces the DosR regulon under normoxic conditions [Fig. 6 and [7]]. These findings suggest that inhibition of respiration (or hypoxia) is not the sole factor that induces the DosR dormancy regulon, and are consistent with the action of the anti-TB drug bedaquiline, which also stimulates respiration [60] and induces the DosR dormancy regulon [61].

Our understanding of how NO [5] and CO [4,5] are sensed directly by DosS and DosT, in a manner dependent on their redox or oxygenation states, respectively, is incomplete. DosS and DosT have different affinities for NO and CO which may, in part, explain this differential response. In contrast, we show that only DosS binds H_2_S; however, our data using *Mtb* Δ*dosS* and Δ*dosT* indicate that upregulation of DosR genes in response to H_2_S also requires DosT. This result was not unexpected, since we observed that induction of DosR regulon genes in Δ*dosT* cells exposed to GYY4137 was similar to that in Δ*dosS* cells [7]. While the mechanism underlying this apparent requirement for DosT in H_2_S-dependent induction of DosR regulon genes is unknown, our data have not ruled out the possibility that DosT can also be activated by sulfide. This is a possibility if DosT(Fe^2+^-O_2_) is autoxidized in the presence of oxygen to DosT(Fe^3+^) within the cellular environment. Overall, caution is warranted when extrapolating DosS and DosT biochemical findings to the mycobacterial cellular environment.

We (Fig. 1B [9]) and others [22,56] have shown that purified DosS exists in the Fe^3+^ state; however, others have reported DosS to be in the ferrous-oxy bound (Fe^2+^-O_2_) form [20,58]. The reasons for this discrepancy remain unclear but are likely due to differences in protein purification methodology and experimental approaches. Nonetheless, our EPR and UV–Vis spectroscopy data provide compelling evidence that DosS heme is in the ferric state (Fig. 1B and F). Also, our data showing that only DosS(Fe^3+^) can bind H_2_S is consistent with numerous studies demonstrating that ferric, but not ferrous heme iron in proteins binds H_2_S [15,16,18,45].

## 4 Conclusions

In light of previous studies showing that *Mtb* infection of macrophages induces upregulation of host H_2_S-producing enzymes CBS [7] and CSE [6], which leads to excessive levels of H_2_S to exacerbate disease, it was important to determine whether *Mtb* senses and responds to physiological levels of H_2_S. Here, we report a mechanism whereby DosS(Fe^3+^) directly binds H_2_S leading to increased DosS autokinase activity and DosR phosphorylation, and that during infection *Mtb* senses endogenous levels of H_2_S to induce key DosR regulon genes. We also show that sulfide binding to DosS(Fe^3+^) slowly reduces the heme iron in the presence of oxygen, which may have implications on DosS activation through subsequent binding NO or CO. Interestingly, we also observed an indirect role for DosT in DosS-mediated transcriptional responses to H_2_S. Overall, the ability of *Mtb* to induce the DosR regulon in response to four physiologically relevant gasotransmitters points to a sophisticated signal transduction system to ensure *Mtb* persistence**.**

## 5 Materials and Methods

**Mycobacterial strains and culturing conditions.***Mtb* H37Rv (BEI Resources, NR-123) and *Mtb* H37Rv *ΔdosS* and *ΔdosT* deletion mutants (provided by Dr. David Sherman, University of Washington) were grown at 37 °C with shaking in Middlebrook 7H9 medium (Difco) supplemented with 10% (vol/vol) ADS (albumin, dextrose, sodium chloride), 0.2% glycerol and 0.02% tyloxapol. Sodium sulfide (Na_2_S.9H_2_O) (Sigma) was added to culture media as an H_2_S donor.

**Expression and Purification of Recombinant DosS and DosT.** DosS and DosT were expressed in Rosetta (DE3) BL21 *E.coli* cells grown at 37 °C in LB medium as reported previously [9]. Briefly, bacterial cells were grown at 37 °C to an OD_600_ of 0.5–0.6, at which point hemin (Sigma) was added to a final concentration of 20 μM hemin and protein production was induced by addition of IPTG to a final concentration of 0.4 mM. Cells were grown overnight at 18 °C, collected by centrifugation, and lysed by sonication in 50 mM sodium phosphate buffer containing 150 mM NaCl, 5% glycerol, pH 7.4. Soluble proteins were extracted by using Profinity IMAC Ni-charged resin (Bio-Rad, Hercules, CA) as recommended by the manufacturer. Following elution, proteins were dialyzed against 50 mM sodium phosphate buffer at 4 °C to remove imidazole.

**Preparation of Sulfide Stock Solutions.** Na_2_S stock solutions (100 mM) were prepared by weighing and dissolving Na_2_S·9H_2_O in argon-deoxygenated ultrapure distilled water (Invitrogen) inside an anaerobic chamber. Working stocks were prepared by diluting stock solutions in argon-deoxygenated 50 mM sodium phosphate buffer (150 mM NaCl, 5% glycerol, pH 7.4) containing 100 μM diethylenetriaminepentaacetic acid (DTPA) as a metal chelator. All Na_2_S stock solutions were made in glass vials with a rubber septum and no headspace. The concentration of total sulfide (H_2_S + HS^−^) in Na_2_S stock solutions was determined by spectrophotometry using DTNB [62,63] and were 6.0 ± 1.5% lower than gravimetric (theoretical) concentrations. When required, the concentration of H_2_S in stock solutions was calculated from the measured pH of sodium phosphate buffer (pH 7.4) given that [H_2_S] = [Na_2_S_DTNB_]/(1 + 10^pH−pKa1^), assuming pKa1 = 7.02 at 25 °C of the equilibrium H_2_S ⇌ HS- + H+ ⇌ S^2−^ + 2H+ [64] [S^2−^] was not considered due to its high pKa2 (14.07) [64]. In 7H9 medium, the concentration of H_2_S was calculated considering culture volume, headspace and Henry's Law constant for H_2_S of 14.62 atm-L/mol at 40 °C [65], 15 mL media in 45 mL vessel at 1 atm headspace pressure, pH = 6.7 and pKa = 6.77 at 37 °C as reported [66].

**UV–Vis Absorption Spectroscopy.** The absorbance spectra of recombinant DosS and DosT (3 μM protein in 50 mM sodium phosphate buffer containing 150 mM NaCl, 5% glycerol, pH 7.4) were determined at room temperature using quartz cuvettes with rubber stoppers in a DU800 spectrophotometer (Beckman Coulter, Fullerton, CA). The reduction of recombinant DosS was achieved by adding sodium dithionite (DTH) to a final concentration of 400 μM using an anaerobic glove box (Plas-Labs Inc., Lansing, MI). A Hamilton syringe with a thin-gauge needle was used to add Na_2_S to protein solutions contained in quartz cuvettes (Spectrocell) sealed with a screw cap containing a rubber septum.

**Determination of K**_**D**_^**app**^**for H**_**2**_**S binding to DosS**. The absorbance spectra of recombinant DosS in the presence of H_2_S were determined at room temperature in 50 mM sodium phosphate buffer containing 150 mM NaCl, 5% glycerol, pH 7.4. The K_D_^app^ for H_2_S binding to DosS was determined by difference spectroscopy using the Soret region. Fractional saturation was determined assuming complete occupancy at 100 μM total sulfide (H_2_S + HS^−^) and was verified by comparing to 200 μM total sulfide.

**EPR Spectroscopy**. Purified DosS protein (10 μM) in sodium phosphate buffer with and without Na_2_S was transferred to thin-walled quartz EPR sample tubes (SP Wilmad-LabGlass, Vineland, NJ) and snap-frozen in liquid nitrogen. Cryogenic (7K) EPR was measured on a Bruker EMX spectrometer operating at a frequency of 9.39 GHz, 15-G modulation amplitude, 33 db power, 81.92-ms time constant, and 41.94-s sweep time. Each sample was scanned 4–8 times and the average taken.

***In Vitro* Phosphorylation Assay.***In vitro* autokinase assays were performed essentially as described [34]**.** Briefly, recombinant DosS (6 μM) alone or in the presence of 100 μM Na_2_S was assayed for its ability to autophosphorylate in a reaction containing 50 μCi of [ γ-^32^P]-labeled ATP (6000 Ci/mmol, PerkinElmer Health Sciences), 100 mM Tris-HCl, pH 7.4, 5 mM MgCl_2_, 50 mM KCl_2_ in a final volume of 20 μl. The reaction was carried out at room temperature, and 4 μl aliquots of reaction mixture were removed from the reaction at various time points between 0 and 60 min. The reaction at each time point was stopped by adding 2X SDS-PAGE sample buffer. The samples were resolved on 4–20% gradient polyacrylamide gels (Bio-Rad) without heating. Resolved proteins were transferred to a PVDF membrane which was exposed to a phosphor screen (Amersham) overnight. The phosphor screen was scanned on a FLA7000IP Typhoon Storage Phosphorimager. Transphosphorylation assays were performed as above, except that DosS and DosR proteins were present in a molar ratio of 1:6, respectively.

**Macrophage infection and isolation of intracellular *Mtb****.* RAW 264.7 macrophage cells (ATCC) were cultured in standard DMEM medium (Gibco) supplemented with 10% heat-inactivated FBS and 10 mM HEPES to maintain pH 7.4. 24 h prior to infection, standard DMEM medium was replaced with methionine/cystine-free DMEM (Gibco). For infection, RAW 264.7 macrophages in 75 cm^2^ flasks were incubated with *Mtb* in log-phase growth at an MOI of 1:5 in methionine/cystine-free DMEM for 2 h. *Mtb*-containing infection media was removed and cells were washed 2X with fresh methionine/cystine-free DMEM. The infected cells were then placed in methionine/cystine-free DMEM supplemented with 10% heat-inactivated FBS and 10 mM HEPES with or without L-Cys (0.1–2 mM) or 400 μM GYY4137. After 24 h, L-Cys- or GYY4137-containing media was removed and cells were washed 1X time with methionine/cystine-free DMEM. Next, intracellular *Mtb* bacilli were isolated essentially as described by Rohde, et al. [67] with a few modifications. Briefly, infected macrophages were lysed in a solution of 4 M guanidine thiocyanate, 0.5% Na N-lauryl sarcosine, 25 mM sodium citrate, and 0.1 M β-mercaptoethanol. Lysate were vortexed and passed through a 21-gauge needle ten times to shear genomic DNA and reduce viscosity. Intracellular mycobacteria were recovered by centrifugation at ∼2700×*g* for 30 min. The bacterial pellet was suspended in 100 μl PBS and lysozyme was added to a final concentration of 0.1 mg/ml an incubated for 30 min at RT. The bacilli were lysed in 1 ml Trizol heated to 65 °C with 0.1 mm Lysing Matrix B silicon beads using a Fastprep-24 bead beater (MP Biomedicals). Next, the lysate was centrifuged to pellet cell debris and the supernatant fraction was treated with 500 μl of chloroform, vortexed, and centrifuged. RNA was isolated from the aqueous layer using a Qiagen RNA isolation kit, following the manufacturer's instructions. RNA was DNase treated prior to qRT-PCR analysis. For macrophage infection assays involving Cys, a negative control group containing bacteria only was exposed to methionine/cystine-free DMEM supplemented with 10% heat-inactivated FBS and 10 mM HEPES pH 7.4 was used to determine baseline gene expression levels.

***Mtb* RNA Isolation and qRT-PCR Analysis.***Mtb* was grown to an OD_600_ of 0.4–0.5, and then Na_2_S was added to a final concentration of 50 μM. After incubation at 37 °C for 30 min, a volume of 4 M guanidine thiocyanate solution equal to the culture volume was added, and cells were collected by centrifugation. The cell pellet was washed 1X time with PBS and then suspended in 1 ml RNAPro solution (MP Biomedicals). The cells were then added to a 2 mL tube containing 0.1 mm Lysing Matrix B beads (MP Biomedicals) and were lysed using a Fastprep-24 bead beater. The lysate was centrifuged to pellet cell debris. 500 μl of chloroform was added to the supernatant fraction which was then vortexed and centrifuged for phase separation. Total RNA was isolated from the aqueous layer using a Qiagen RNA isolation kit, following the manufacturer's instructions. RNA was treated with DNaseI prior to qRT-PCR analysis. 500 ng of DNaseI-treated RNA was used for cDNA synthesis using the iScript cDNA synthesis kit (Bio-Rad, USA). Quantitative RT-PCR was performed using SsoAdvanced SYBR green supermix (Bio-Rad, USA) with the Bio-Rad CFX96 detection system according to the manufacturer's instructions. Quantitative RT-PCR reactions were performed in duplicate using three independent *Mtb* cultures. Relative changes in gene expression were determined using the 2ΔCt method [68], where Ct values of target genes were normalized to Ct values of *Mtb sigA* mRNA. Relative changes are shown as the ratio of expression of genes between Na_2_S-exposed and untreated bacterial cultures, or between *Mtb* isolated from RAW 264.7 macrophages grown in cysteine/methionine-free medium and *Mtb* isolated from macrophages grown the same medium containing 200 μM cysteine or 400 μM GYY4137. Primers used in this study are listed in Table S3.

**Detection of Intracellular H**_**2**_**S Using the WSP-5 Probe.** The H_2_S-specfic WSP-5 fluorescent probe (CAS # 1593024-78-2) was prepared according to the manufacturer's instructions. Cell staining was performed as described [37] with some modifications. Briefly, 5 × 10^4^ RAW 264.7 macrophages were plated in each well of 96 well plate and incubated overnight in DMEM medium containing 0.1 - 2.0 mM l-cysteine. The next day the medium was removed, and fresh medium containing 100 μM CTAB, the desired l-cysteine concentration, and 50 μM WSP-5 probe was added. After 30 min, the WSP-5-containing medium was removed, the cells were washed once with PBS and placed in fresh PBS for imaging. Fluorescence imaging was performed using Biotek Cytation 5 plate reader.

## 6 Computational methods

**Starting Structure.** The starting structure of the DosS heme-containing GAF-A domain was built from the corresponding X-ray structure (PDB entry code 2w3e) [22]. Protonation states of amino acids were those corresponding to their physiological state at neutral pH (i.e., Asp and Glu negatively charged, Lys and Arg positively charged). His149, corresponding to the heme proximal ligand, was simulated in the HID state (with protonated N δ). The remaining His residues were simulated favoring H-bond formation. Particular care was taken for His^89^ which is part of the distal H-bond network. Since the template crystal structure consists of a truncated GAF-A domain, a C-terminal carboxyl group is present that is not present in the full structure. To account for this, a N–CH_3_ “cap” was added to this carboxyl groups to avoid interaction with nearby positive residues. The system was solvated by constructing an octahedral box of 10–12 Å using AmberTools.

**Classical Molecular Dynamics (MD) Simulations.** The MD simulations were performed using the Assisted Model Building with Energy Refinement (AMBER) package [69]. Heme, as well as bound and free H_2_S and HS^−^ parameters, were taken from our previous work related to H_2_S/HS^−^ binding to heme proteins [28]. All MD simulations were performed using periodic boundary conditions, SHAKE algorithm and the particle mesh Ewald (PME) summation method for treating the electrostatic interactions using default AMBER 16 parameters [69]. Each system was first optimized, and then slowly equilibrated to reach proper temperature and pressure values using the Langevin thermostat and Berendsen barostat [70]. All four truncated endpoints of the protein (exposed to solvent) were simulated with mild restraints applied to them to maintain α-helix structure.

**Ligand Binding Free Energy Profiles.** To determine H_2_S/HS^−^ binding free energy profiles, we used our previously developed and extensively used Steered MD (sMD) approach combined with Jarzynski's equation [71,72]. Briefly, in each simulation the ligand is pulled towards the heme iron inside the protein active site using a harmonic guiding potential at constant speed, and the corresponding work performed on the system is recorded. Several simulations are performed starting from different initial conformations with the ligand outside the protein, and finally the corresponding free energy profile is obtained combining the corresponding work profiles using Jarzynski's equality. We employed 98 different trajectories for H_2_S and 89 for HS^−^. In all cases, the guiding coordinate was the Fe–S distance, using a force constant of 200 kcal/mol.Å^2^ and a speed of 0.0025 Å/ps.

**Hybrid Quantum Mechanics/Molecular Mechanics Simulations.** QM/MM simulations were performed with our own extensively tested and developed code, called LIO which combines a Gaussian-based density functional theory (DFT) approach implemented in CUDA with the AMBER force field and is implemented as a QM/MM option in AMBER [73]. System parameters were the same as in our previous QM/MM work on heme proteins [26,28]. Briefly, the heme, its proximal and distal ligands, a nearby water molecule and a heme propionate define the QM subsystem, and the remaining protein and solvent atoms the classical system. Covalent bonds between QM and MM subsystems were treated using the Link atom method [74]. The QM system is simulated using a double Z plus polarization basis set [75] and the PBE exchange-correlation functional [76]. Heme iron in the Fe^3+^ state bound to H_2_S/HS^−^ was simulated in the low-spin state. We performed a 2.0 ps MD simulation, starting from a snapshot extracted from the previously optimized MD simulation.

## Author contributions

Conceptualization and Design: RRS, JRL and AJCS. Execution: UV–Vis studies and biochemical assays (RRS), EPR studies (SB, EA and DBKS), MD modeling (MP, MM, DE), *in vitro* macrophage assays (RRS, VPR). Data analysis: RRS, JRL, JNG, VPR, DBKS, MM, DE and AJCS. Technical expertise: JRL, DBSK, MP, MM, DE. Writing: RRS, JNG and AJCS wrote the manuscript. Editing: JRL, JNG, DE and AJCS. Figure preparation: RRS. All authors discussed the results and commented on the manuscript.

## Declaration of competing interest

The authors declare no conflicts of interest.

## References

[1] (2020). Global Tuberculosis Report 2020.

[2] Chinta K.C., Rahman M.A., Saini V., Glasgow J.N., Reddy V.P., Lever J.M., Nhamoyebonde S., Leslie A., Wells R.M., Traylor A., Madansein R., Siegal G.P., Antony V.B., Deshane J., Wells G., Nargan K., George J.F., Ramdial P.K., Agarwal A., Steyn A.J.C. (2018). Microanatomic distribution of myeloid heme oxygenase-1 protects against free radical-mediated immunopathology in human tuberculosis. Cell Rep..

[3] Nicholson S., Bonecini-Almeida Mda G., Lapa e Silva J.R., Nathan C., Xie Q.W., Mumford R., Weidner J.R., Calaycay J., Geng J., Boechat N., Linhares C., Rom W., Ho J.L. (1996). Inducible nitric oxide synthase in pulmonary alveolar macrophages from patients with tuberculosis. J. Exp. Med..

[4] Kumar A., Deshane J.S., Crossman D.K., Bolisetty S., Yan B.S., Kramnik I., Agarwal A., Steyn A.J. (2008). Heme oxygenase-1-derived carbon monoxide induces the Mycobacterium tuberculosis dormancy regulon. J. Biol. Chem..

[5] Shiloh M.U., Manzanillo P., Cox J.S. (2008). Mycobacterium tuberculosis senses host-derived carbon monoxide during macrophage infection. Cell Host Microbe.

[6] Rahman M.A., Cumming B.M., Addicott K.W., Pacl H.T., Russell S.L., Nargan K., Naidoo T., Ramdial P.K., Adamson J.H., Wang R., Steyn A.J.C. (2020). Hydrogen sulfide dysregulates the immune response by suppressing central carbon metabolism to promote tuberculosis. Proc. Natl. Acad. Sci. U. S. A..

[7] Saini V., Chinta K.C., Reddy V.P., Glasgow J.N., Stein A., Lamprecht D.A., Rahman M.A., Mackenzie J.S., Truebody B.E., Adamson J.H., Kunota T.T.R., Bailey S.M., Moellering D.R., Lancaster J.R., Steyn A.J.C. (2020). Hydrogen sulfide stimulates Mycobacterium tuberculosis respiration, growth and pathogenesis. Nat. Commun..

[8] Kinger A.K., Tyagi J.S. (1993). Identification and cloning of genes differentially expressed in the virulent strain of Mycobacterium tuberculosis. Gene.

[9] Kumar A., Toledo J.C., Patel R.P., Lancaster J.R., Steyn A.J. (2007). Mycobacterium tuberculosis DosS is a redox sensor and DosT is a hypoxia sensor. Proc. Natl. Acad. Sci. U. S. A..

[10] Voskuil M.I., Schnappinger D., Visconti K.C., Harrell M.I., Dolganov G.M., Sherman D.R., Schoolnik G.K. (2003). Inhibition of respiration by nitric oxide induces a Mycobacterium tuberculosis dormancy program. J. Exp. Med..

[11] Kunota T.T.R., Rahman M.A., Truebody B.E., Mackenzie J.S., Saini V., Lamprecht D.A., Adamson J.H., Sevalkar R.R., Lancaster J.R., Berney M., Glasgow J.N., Steyn A.J.C. (2021).

[12] Fu L., Liu K., He J., Tian C., Yu X., Yang J. (2020). Direct proteomic mapping of cysteine persulfidation. Antioxidants Redox Signal..

[13] Filipovic M.R., Zivanovic J., Alvarez B., Banerjee R. (2018). Chemical biology of H2S signaling through persulfidation. Chem. Rev..

[14] Kumar R., Banerjee R. (2021). Regulation of the redox metabolome and thiol proteome by hydrogen sulfide. Crit. Rev. Biochem. Mol. Biol..

[15] Keilin F.R.S.D. (1933). On the combination of Methaemoglobin with H_2_S. Proc. Roy. Soc. Lond..

[16] Jensen B., Fago A. (2018). Reactions of ferric hemoglobin and myoglobin with hydrogen sulfide under physiological conditions. J. Inorg. Biochem..

[17] Kraus D.W., Wittenberg J.B., Lu J.F., Peisach J. (1990). Hemoglobins of the Lucina pectinata/bacteria symbiosis. II. An electron paramagnetic resonance and optical spectral study of the ferric proteins. J. Biol. Chem..

[18] Mot A.C., Bischin C., Damian G., Attia A.A.A., Gal E., Dina N., Leopold N., Silaghi-Dumitrescu R. (2018). Fe(III) - sulfide interaction in globins: characterization and quest for a putative Fe(IV)-sulfide species. J. Inorg. Biochem..

[19] Pietri R., Lewis A., Leon R.G., Casabona G., Kiger L., Yeh S.R., Fernandez-Alberti S., Marden M.C., Cadilla C.L., Lopez-Garriga J. (2009). Factors controlling the reactivity of hydrogen sulfide with hemeproteins. Biochemistry.

[20] Sousa E.H., Tuckerman J.R., Gonzalez G., Gilles-Gonzalez M.A. (2007). DosT and DevS are oxygen-switched kinases in Mycobacterium tuberculosis. Protein Sci..

[21] Smith D.W., Williams R.J.P., Hemmerich P. (1970).

[22] Cho H.Y., Cho H.J., Kim Y.M., Oh J.I., Kang B.S. (2009). Structural insight into the heme-based redox sensing by DosS from Mycobacterium tuberculosis. J. Biol. Chem..

[23] Morris K.Y. (1972). Kinetics of oxidation of aqueous sulfide by O_2_. Environ. Sci. Technol..

[24] Peisach J., Blumberg W.E., Ogawa S., Rachmilewitz E.A., Oltzik R. (1971). The effects of protein conformation on the heme symmetry in high spin ferric heme proteins as studied by electron paramagnetic resonance. J. Biol. Chem..

[25] Takashi H.R.D., John Y., Leigh S., Reed George H., Water-Man Michael R., Asakura Toshio (1970). Electromagnetic properties of hemoproteins. J. Biol. Chem..

[26] Madrona Y., Waddling C.A., Ortiz de Montellano P.R. (2016). Crystal structures of the CO and NOBound DosS GAF-A domain and implications for DosS signaling in Mycobacterium tuberculosis. Arch. Biochem. Biophys..

[27] Boubeta F.M., Bieza S.A., Bringas M., Estrin D.A., Boechi L., Bari S.E. (2018). Mechanism of sulfide binding by ferric hemeproteins. Inorg. Chem..

[28] Boubeta F.M., Bari S.E., Estrin D.A., Boechi L. (2016). Access and binding of H2S to hemeproteins: the case of HbI of lucina pectinata. J. Phys. Chem. B.

[29] Bikiel D.E., Boechi L., Capece L., Crespo A., De Biase P.M., Di Lella S., Gonzalez Lebrero M.C., Marti M.A., Nadra A.D., Perissinotti L.L., Scherlis D.A., Estrin D.A. (2006). Modeling heme proteins using atomistic simulations. Phys. Chem. Chem. Phys..

[30] Capece L., Boechi L., Perissinotti L.L., Arroyo-Manez P., Bikiel D.E., Smulevich G., Marti M.A., Estrin D.A. (2013). Small ligand-globin interactions: reviewing lessons derived from computer simulation. Biochim. Biophys. Acta.

[31] Vitvitsky V., Yadav P.K., Kurthen A., Banerjee R. (2015). Sulfide oxidation by a noncanonical pathway in red blood cells generates thiosulfate and polysulfides. J. Biol. Chem..

[32] Bostelaar T., Vitvitsky V., Kumutima J., Lewis B.E., Yadav P.K., Brunold T.C., Filipovic M., Lehnert N., Stemmler T.L., Banerjee R. (2016). Hydrogen sulfide oxidation by myoglobin. J. Am. Chem. Soc..

[33] Bianco C.L., Savitsky A., Feelisch M., Cortese-Krott M.M. (2018). Investigations on the role of hemoglobin in sulfide metabolism by intact human red blood cells. Biochem. Pharmacol..

[34] Roberts D.M., Liao R.P., Wisedchaisri G., Hol W.G., Sherman D.R. (2004). Two sensor kinases contribute to the hypoxic response of Mycobacterium tuberculosis. J. Biol. Chem..

[35] Abe K., Kimura H. (1996). The possible role of hydrogen sulfide as an endogenous neuromodulator. J. Neurosci..

[36] Stipanuk M.H., Beck P.W. (1982). Characterization of the enzymic capacity for cysteine desulphhydration in liver and kidney of the rat. Biochem. J..

[37] Peng B., Chen W., Liu C., Rosser E.W., Pacheco A., Zhao Y., Aguilar H.C., Xian M. (2014). Fluorescent probes based on nucleophilic substitution-cyclization for hydrogen sulfide detection and bioimaging. Chemistry.

[38] Brigham M.P., Stein W.H., Moore S. (1960). The concentrations of cysteine and cystine in human blood plasma. J. Clin. Investig..

[39] Goodman M.T., McDuffie K., Hernandez B., Wilkens L.R., Selhub J. (2000). Case-control study of plasma folate, homocysteine, vitamin B12, and cysteine as markers of cervical dysplasia. Cancer.

[40] Murphy G., Fan J.-H., Mark S.D., Dawsey S.M., Selhub J., Wang J., Taylor P.R., Qiao Y.-L., Abnet C.C. (2011). Prospective study of serum cysteine levels and oesophageal and gastric cancers in China. Gut.

[41] Park H.D., Guinn K.M., Harrell M.I., Liao R., Voskuil M.I., Tompa M., Schoolnik G.K., Sherman D.R. (2003). Rv3133c/dosR is a transcription factor that mediates the hypoxic response of Mycobacterium tuberculosis. Mol. Microbiol..

[42] Dilek N., Papapetropoulos A., Toliver-Kinsky T., Szabo C. (2020). Hydrogen sulfide: an endogenous regulator of the immune system. Pharmacol. Res..

[43] Wang R. (2012). Physiological implications of hydrogen sulfide: a whiff exploration that blossomed. Physiol. Rev..

[44] Fojtikova V., Bartosova M., Man P., Stranava M., Shimizu T., Martinkova M. (2016). Effects of hydrogen sulfide on the heme coordination structure and catalytic activity of the globin-coupled oxygen sensor AfGcHK. Biometals.

[45] Takahashi H., Sekimoto M., Tanaka M., Tanaka A., Igarashi J., Shimizu T. (2012). Hydrogen sulfide stimulates the catalytic activity of a heme-regulated phosphodiesterase from Escherichia coli (Ec DOS). J. Inorg. Biochem..

[46] Belton M., Brilha S., Manavaki R., Mauri F., Nijran K., Hong Y.T., Patel N.H., Dembek M., Tezera L., Green J., Moores R., Aigbirhio F., Al-Nahhas A., Fryer T.D., Elkington P.T., Friedland J.S. (2016). Hypoxia and tissue destruction in pulmonary TB. Thorax.

[47] Mehra S., Foreman T.W., Didier P.J., Ahsan M.H., Hudock T.A., Kissee R., Golden N.A., Gautam U.S., Johnson A.M., Alvarez X., Russell-Lodrigue K.E., Doyle L.A., Roy C.J., Niu T., Blanchard J.L., Khader S.A., Lackner A.A., Sherman D.R., Kaushal D. (2015). The DosR regulon modulates adaptive immunity and is essential for Mycobacterium tuberculosis persistence. Am. J. Respir. Crit. Care Med..

[48] Gautam U.S., McGillivray A., Mehra S., Didier P.J., Midkiff C.C., Kissee R.S., Golden N.A., Alvarez X., Niu T., Rengarajan J., Sherman D.R., Kaushal D. (2015). DosS Is required for the complete virulence of mycobacterium tuberculosis in mice with classical granulomatous lesions. Am. J. Respir. Cell Mol. Biol..

[49] Gautam U.S., Mehra S., Kumari P., Alvarez X., Niu T., Tyagi J.S., Kaushal D. (2019). Mycobacterium tuberculosis sensor kinase DosS modulates the autophagosome in a DosR-independent manner. Commun. Biol..

[50] Rustad T.R., Harrell M.I., Liao R., Sherman D.R. (2008). The enduring hypoxic response of Mycobacterium tuberculosis. PLoS One.

[51] Pietri R., Roman-Morales E., Lopez-Garriga J. (2011). Hydrogen sulfide and hemeproteins: knowledge and mysteries. Antioxidants Redox Signal..

[52] Fukuto J.M., Ignarro L.J., Nagy P., Wink D.A., Kevil C.G., Feelisch M., Cortese-Krott M.M., Bianco C.L., Kumagai Y., Hobbs A.J., Lin J., Ida T., Akaike T. (2018). Biological hydropersulfides and related polysulfides - a new concept and perspective in redox biology. FEBS Lett..

[53] Basudhar D., Madrona Y., Yukl E.T., Sivaramakrishnan S., Nishida C.R., Moenne-Loccoz P., Ortiz de Montellano P.R. (2016). Distal hydrogen-bonding interactions in ligand sensing and signaling by Mycobacterium tuberculosis DosS. J. Biol. Chem..

[54] Yukl E.T., Ioanoviciu A., Nakano M.M., de Montellano P.R., Moenne-Loccoz P. (2008). A distal tyrosine residue is required for ligand discrimination in DevS from Mycobacterium tuberculosis. Biochemistry.

[55] Cho H.Y., Cho H.J., Kim M.H., Kang B.S. (2011). Blockage of the channel to heme by the E87 side chain in the GAF domain of Mycobacterium tuberculosis DosS confers the unique sensitivity of DosS to oxygen. FEBS Lett..

[56] Zheng H., Colvin C.J., Johnson B.K., Kirchhoff P.D., Wilson M., Jorgensen-Muga K., Larsen S.D., Abramovitch R.B. (2017). Inhibitors of Mycobacterium tuberculosis DosRST signaling and persistence. Nat. Chem. Biol..

[57] Ely F., Nunes J.E., Schroeder E.K., Frazzon J., Palma M.S., Santos D.S., Basso L.A. (2008). The Mycobacterium tuberculosis Rv2540c DNA sequence encodes a bifunctional chorismate synthase. BMC Biochem..

[58] Ioanoviciu A., Meharenna Y.T., Poulos T.L., Ortiz de Montellano P.R. (2009). DevS oxy complex stability identifies this heme protein as a gas sensor in Mycobacterium tuberculosis dormancy. Biochemistry.

[59] Voskuil M.I., Visconti K.C., Schoolnik G.K. (2004). Mycobacterium tuberculosis gene expression during adaptation to stationary phase and low-oxygen dormancy. Tuberculosis.

[60] Mackenzie J.S., Lamprecht D.A., Asmal R., Adamson J.H., Borah K., Beste D.J.V., Lee B.S., Pethe K., Rousseau S., Krieger I., Sacchettini J.C., Glasgow J.N., Steyn A.J.C. (2020). Bedaquiline reprograms central metabolism to reveal glycolytic vulnerability in Mycobacterium tuberculosis. Nat. Commun..

[61] Koul A., Vranckx L., Dhar N., Gohlmann H.W., Ozdemir E., Neefs J.M., Schulz M., Lu P., Mortz E., McKinney J.D., Andries K., Bald D. (2014). Delayed bactericidal response of Mycobacterium tuberculosis to bedaquiline involves remodelling of bacterial metabolism. Nat. Commun..

[62] Nagy P., Palinkas Z., Nagy A., Budai B., Toth I., Vasas A. (2014). Chemical aspects of hydrogen sulfide measurements in physiological samples. Biochim. Biophys. Acta.

[63] Nashef A.S., Osuga D.T., Feeney R.E. (1977). Determination of hydrogen sulfide with 5,5'-dithiobis-(2-nitrobenzoic acid), N-ethylmaleimide, and parachloromercuribenzoate. Anal. Biochem..

[64] Li Q., Lancaster J.R. (2013). Chemical foundations of hydrogen sulfide biology. Nitric Oxide.

[65] Rinker E.B., Sandall O.C. (2000). Physical solubility of hydrogen sulfide in several aqueous solvents. Can. J. Chem. Eng..

[66] Olson K.R. (2012). A practical look at the chemistry and biology of hydrogen sulfide. Antioxidants Redox Signal..

[67] Rohde K.H., Abramovitch R.B., Russell D.G. (2007). Mycobacterium tuberculosis invasion of macrophages: linking bacterial gene expression to environmental cues. Cell Host Microbe.

[68] Schmittgen T.D., Livak K.J. (2008). Analyzing real-time PCR data by the comparative C(T) method. Nat. Protoc..

[69] K.B. D.A. Case, I.Y. Ben-Shalom, S.R. Brozell, D.S. Cerutti, T.E. Cheatham, III, V.W.D. Cruzeiro, T.A. Darden, R.E. Duke, G. Giambasu, M.K. Gilson, H. Gohlke, A.W. Goetz, R. Harris, S. Izadi, S.A. Izmailov, K. Kasavajhala, A. Kovalenko, R. Krasny, T. Kurtzman, T.S. Lee, S. LeGrand, P. Li, C. Lin, J. Liu, T. Luchko, R. Luo, V. Man, K.M. Merz, Y. Miao, O. Mikhailovskii, G. Monard, H. Nguyen, A. Onufriev, F.Pan, S. Pantano, R. Qi, D.R. Roe, A. Roitberg, C. Sagui, S. Schott-Verdugo, J. Shen, C.L. Simmerling, N.R.Skrynnikov, J. Smith, J. Swails, R.C. Walker, J. Wang, L. Wilson, R.M. Wolf, X. Wu, Y. Xiong, Y. Xue, D.M. York and P.A. Kollman AMBER 2020.

[70] Berendsen H.J.C.P., J. P M., van Gunsteren W.F., DiNola A., Haak J.R. (1984). Molecular dynamics with coupling to an external bath. J. Chem. Phys..

[71] Arrar M., Boubeta F.M., Szretter M.E., Sued M., Boechi L., Rodriguez D. (2019). On the accurate estimation of free energies using the jarzynski equality. J. Comput. Chem..

[72] Jarzynski C. (1997). Nonequilibrium equality for free energy differences. Phys. Rev. Lett..

[73] Marcolongo J.P., Zeida A., Semelak J.A., Foglia N.O., Morzan U.N., Estrin D.A., Gonzalez Lebrero M.C., Scherlis D.A. (2018). Chemical reactivity and spectroscopy explored from QM/MM molecular dynamics simulations using the LIO code. Front. Chem..

[74] Tavan M.E.a.P. (1999). A hybrid method for solutes in complex solvents: density functional theory combined with empirical force fields. J. Chem. Phys..

[75] Godbout N., Salahub D.R., Andzelm J., Wimmer E. (1992). Optimization of Gaussian-type basis sets for local spin density functional calculations. Part I. Boron through neon, optimization technique and validation. Can. J. Chem..

[76] Perdew J.P., Burke K., Ernzerhof M. (1996). Generalized gradient approximation made simple. Phys. Rev. Lett..

