# Supplementary material for: Mycobacterium tuberculosis DosS binds H2S through its Fe3+ heme iron to regulate the DosR dormancy regulon
Source: Redox Biol. 2022 Apr 20;52:102316. doi: 10.1016/j.redox.2022.102316 (PMC9062744; doi:10.1016/j.redox.2022.102316)
Supplement: Multimedia component 4 [file mmc4.pdf]

Supplementary Information for

Mycobacterium tuberculosis DosS binds H_2S through its Fe^{3+} heme iron to regulate the Dos dormancy regulon

Ritesh R. Sevalkar[a], Joel N. Glasgow[a,1], Martín Pettinati[b,c], Marcelo A. Marti[d,e], Vineel P. Reddy[a], Swati Basu[f], Elmira Alipour[f], Daniel B. Kim-Shapiro[f], Dario A. Estrin[b,c], Jack R. Lancaster, Jr.[g] and Adrie J.C. Steyn[a,h,1]

[a]Department of Microbiology, and Centers for AIDS Research and Free Radical Biology, University of Alabama at Birmingham, Birmingham, AL
[b]Universidad de Buenos Aires, Facultad de Ciencias Exactas y Naturales, Departamento de Química Inorgánica, Analítica y Química Física, Buenos Aires, Argentina
[c]CONICET-Universidad de Buenos Aires, Instituto de Química Física de los Materiales, Medio Ambiente y Energía (INQUIMAE), Buenos Aires, Argentina
[d]Universidad de Buenos Aires, Facultad de Ciencias Exactas y Naturales, Departamento de Química Biológica, Buenos Aires, Argentina
[e]CONICET-Universidad de Buenos Aires, Instituto de Química Biológica (IQUIBICEN), Buenos Aires, Argentina
[f]Department of Physics, Wake Forest University, Winston-Salem, NC
[g]Department of Pharmacology & Chemical Biology, Vascular Medicine Institute, University of Pittsburgh School of Medicine, Pittsburgh, PA
[h]Africa Health Research Institute, University of KwaZulu-Natal, Durban, South Africa

[1]**Corresponding authors**:

Adrie JC Steyn, PhD

Professor, University of Alabama at Birmingham

Investigator, Africa Health Research Institute

Email: asteyn@uab.edu or adrie.steyn@ahri.org

Joel N. Glasgow, PhD
Assistant Professor, University of Alabama at Birmingham
Email: jng@uab.edu

This PDF file includes:

Figures S1 to S6
Table S1-S3
Legends for Movies S1-S3
SI References

Other supplementary materials for this manuscript include the following:

Movies S1-S3

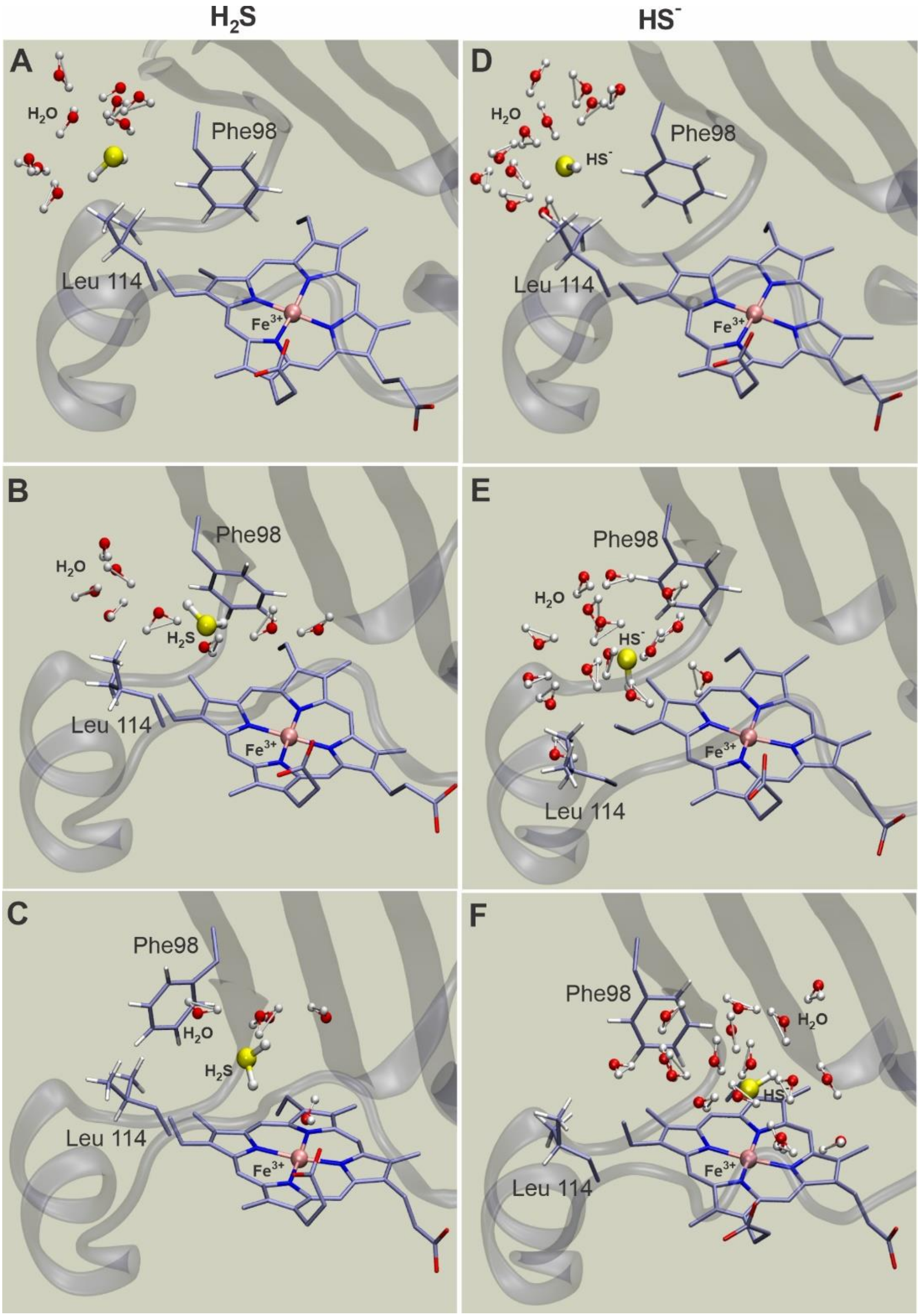

Fig. S1. Steered Molecular Dynamics (sMD) modeling of sulfide entry into the DosS heme pocket. Typical snapshots of H_2S (A-C), and HS^- (D-F) along the entry path into the DosS heme pocket, representing sulfide ligands at a distance of 11, 8 and 5 Å (upper, medium, and lower panels, respectively) from the heme iron.

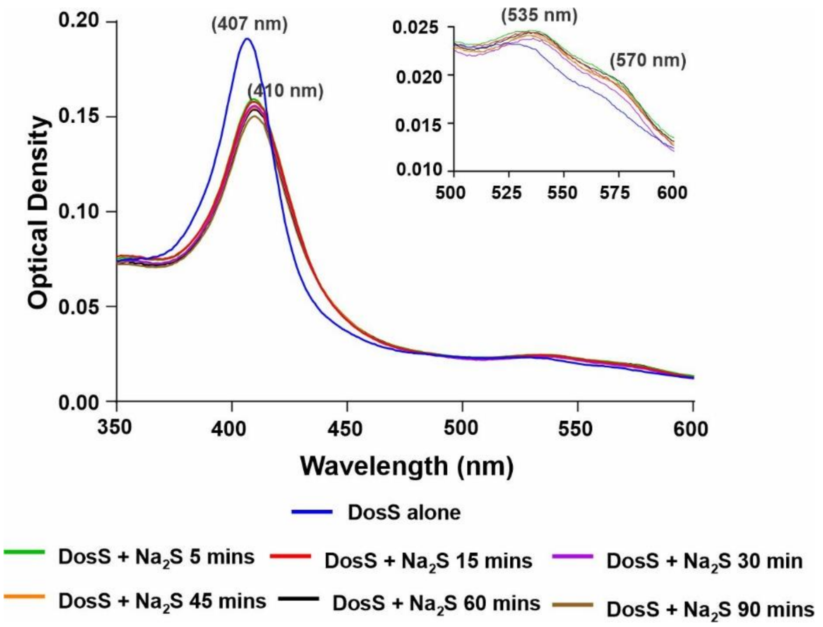

Fig. S2. UV-Vis time-course scan of DosS and Na_2S: Representative UV-Visible spectra of the Fe^{3+} form of DosS (3 µM) for 5-90 minutes following the addition of 30 µM Na_2S. (*Inset*) Absorption spectra replotted to highlight the α (570 nm) and β (535 nm) peaks.

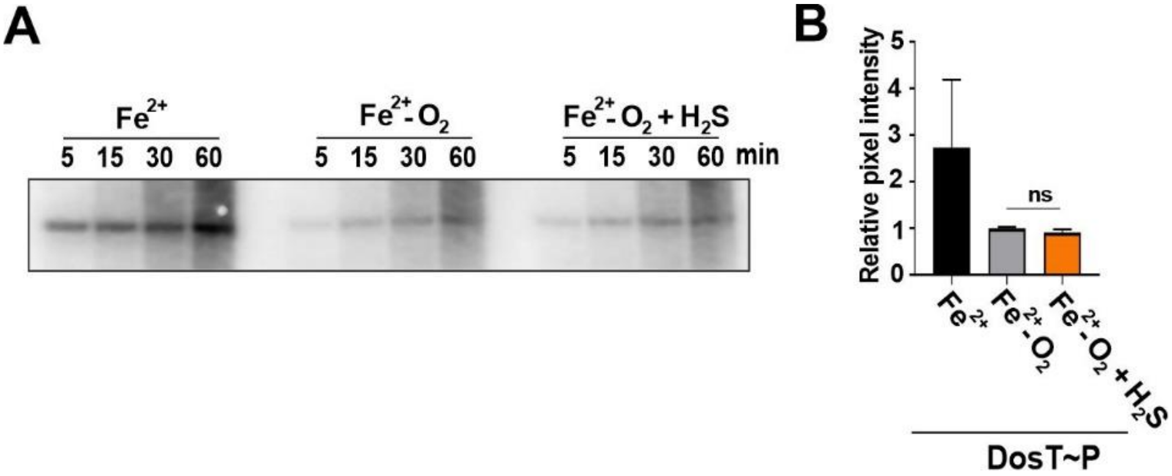

Fig. S3. Autophosphorylation assay. (A) Representative autoradiogram of PAGE-resolved γ-32P-labeled recombinant DosT following autophosphorylation in the presence of γ-32P-ATP alone (left and center panels) or with γ-32P-ATP in the presence of 100 μM Na2S (right panel). (B) Densitometric quantitation of DosS bands in (A) at 60 min (n=3).

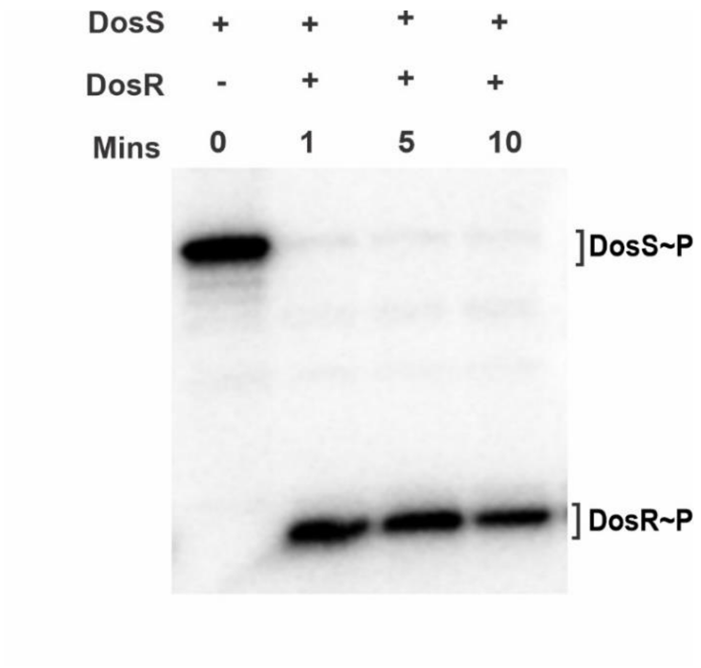

Fig. S4. Transphosphorylation assay. Autoradiogram of PAGE-resolved ^{32}P-labeled DosS in the Fe^{3+} form alone after 60 min of autophosphorylation (left lane), and following addition of recombinant DosR to the autokinase reaction. The reactions were stopped 1-10 min following addition of DosR.

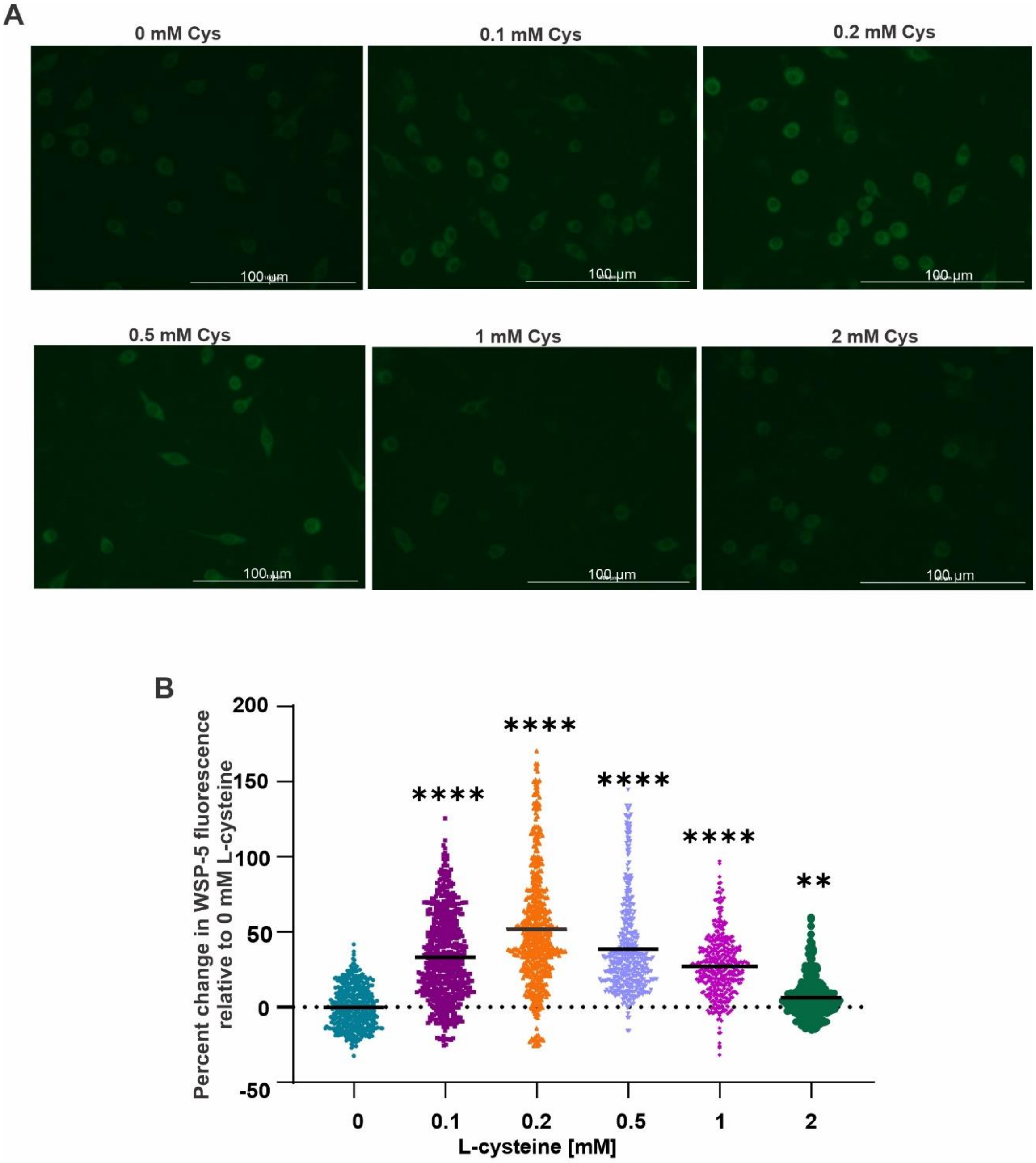

Fig. S5. **Detection of intracellular H_2S.** (A) Representative images of intracellular WSP-5 fluorescence in RAW 264.7 macrophages grown in media containing 0-2.0 mM L-cysteine (Cys). (B) Percent change in intracellular H_2S-dependent probe fluorescence levels in RAW 264.7 macrophages in presence of 0-2.0 mM L-cysteine as determined by WSP-5 fluorescence measurements in individual cells. The data were analyzed using Kruskal-Wallis test with Holm-Sidak multiple comparisons. The solid black line represents the mean value of the group. **$P < 0.01$ and ****$P < 0.0001$ vs 0 mM L-cysteine control.

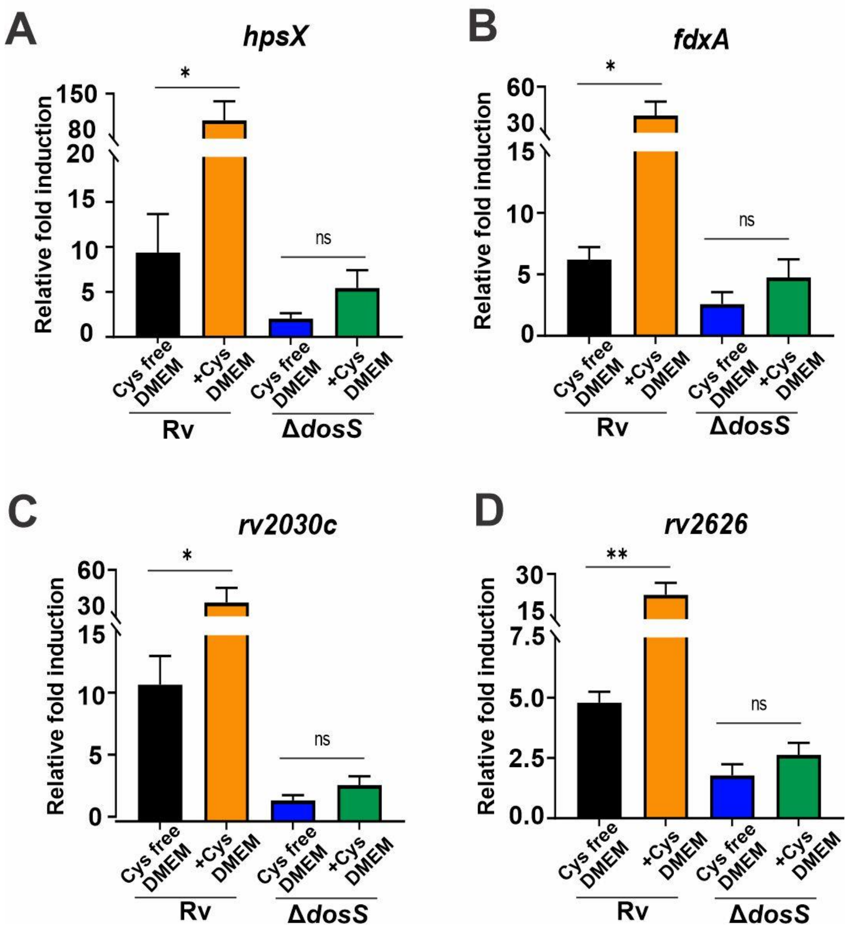

Fig. S6. Effect of Cysteine depletion in DMEM culture on expression of DosR regulon genes. (A-D) Expression of representative Dos regulon genes in WT and ΔdosS *Mtb* isolated from infected RAW 264.7 macrophages grown cysteine-free DMEM or DMEM containing 200 µM L-Cys for 24 hours (n=3). RT-qPCR gene expression data from macrophage-isolated *Mtb* are shown relative to gene expression in WT and ΔdosS *Mtb* cultures exposed to DMEM. Data are shown as the mean ± SEM and were analyzed using a one-way ANOVA with Tukey post-hoc test performed using GraphPad Prism version 9. *$p < 0.05$ and **$P < 0.01$.

Table S1: Comparison of Biophysical Parameters of Sulfide-Binding Proteins

Protein	**UV-vis** (nm)	**EPR** (g-values)	**K_D** (µM)	Reference
Mtb DosS	415, 535, 570 (s)	2.67, 2.26, 1.76	5.64 (H_2S)	This work
L. pectinata Hb I	425, 545, 573 (s)	2.67, 2.24, 1.84	0.09 (XS_T)	(1)
Human Hemoglobin	423, 577, 541	2.51, 2.25, 1.86	17 (XS_T)	(2)
Bovine Hemoglobin	425, 542, 575(s)	2.55, 2.26, 1.88	7(XS_T)	(3)
Equine Myoglobin	427, 547, 580	2.56, 2.25, 1.83	96 (XS_T)	(4), (5)
Human Myeloperoxidase	432, 625	2.567, 2.274, 1.850; 2.512, 2.262, 1.875	< 12 (XS_T)	(6)
Microperoxidase	414, 536, 568	--	219 (calc) (XS_T)	(7)
Truncated *B. subtilis* Hb	427, 550, 577	--	0.2 (XS_T)	(8)
Truncated *T. fusca* Hb	425, 550, 575	--	0.36 (XS_T)	(8)
E. coli DOS (heme-regulated TCS PDE)	427,546, 579	--	ca. 200 (XS_T) (based on PDE activation)	(9)
Anaeromyxobacter AfGcHK (heme-regulated TCS His kinase)	423-426,549-551	--	200-5000 (XS_T) for binding, although no autokinase activation with 10 mM	(10)

Note: Hemeproteins that get reduced on sulfide binding do not show the EPR spectrum and hence were not used for comparison eg., cytochrome c oxidase, neuroglobin, cytochrome c.
H_2S = based on H_2S concentration
XS_T = based on concentration of total sulfide species ($H_2S + HS^- + S^{2-}$)
TCS = two-component system
(s) = shoulder
calc = calculated

Table S2. Comparison of structural and electronic parameters of H_2S and HS^- bound states obtained from QM/MM calculations

Parameter	H_2S	HS^-
dFe-S	2.35 Å	2.17 Å
H_2S or HS^- Mulliken population	0.5543	0.0984
dFe-NHis	2.06 Å	2.09 Å
dS-H1<br>dS-H2	towards Tyr 1.36 Å<br>towards water1.39 Å	1.35 Å
H1 Mulliken population<br>H2 Mulliken population	towards Tyr 0.2242<br>towards water 0.2644	S-H 0.1698<br>Tyr-H 0.4458

Table S3. DNA Primers used for qRT-PCR.

Primer	Primer Sequence (5' to 3')
SigA-For	TCGGTTCGCGCCTACCT
SigA-Rev	TGGCTAGCTCGACCTCTTCCT
hspX-For	CGCACCGAGCAGAAGGAC
hsp-Rev	CCGCCACCGACACAGTAA
fdxA-For	CCTATGTGATCGGTAGTGA
fdxA-Rev	GGGTTGATGTAGAGCATT
rv2030-For	GAATAGTGGTGTGGGCTCATAA
rv2030-Rev	CGTATCGCTCACGGACTATCT
rv2626-For	CGACCGCGACATTGTGAT
rv2626-Rev	CATCGACGTAGTAGATGCTGT

movie_sh2_lado.mp4

Movie S1. Steered Molecular Dynamics modeling of H_2S entry into the DosS heme pocket. The trajectory of H_2S [shown in yellow (S) and white (H) spheres] approaching the DosS heme is shown. H_2S first approaches the Y97-F98 pair, and then passes between F98 and the L114-P115 pair to enter the heme pocket, where it interacts with distal Y171. The H_2S solvation shell waters are represented as small red and white triangles. The overall DosS secondary structure is shown as a cyan tube. Red = oxygen, Blue = nitrogen, Pink = heme center. Movie represents 2.8 ns of simulation with a ligand pulling speed of 0.0025 Â/ps.

movie_sh_lado.mp4

Movie S2. Steered Molecular Dynamics modeling of HS^- entry into the DosS heme pocket. The trajectory of HS^- [shown in yellow (S) and white (H) spheres] approaching the DosS heme is shown. HS^- first approaches the Y97-F98 pair. The barrier for HS^- entry is formed by disrupting the F98-L114 interaction, which is clearly seen when the L114 sidechain changes separates from F98. HS^- can then pass between F98 and the L114-P115 pair to enter the heme pocket, where it interacts with distal Y171. The larger barrier for HS^- is due to the fact that while HS^- enters almost dry, the negatively charged ligand is always solvated and accompanied by several water molecules. The HS^- solvation shell waters are represented as small red and white triangles. The overall DosS secondary structure is shown as a cyan tube. Red = oxygen, Blue = nitrogen, Pink = heme center. Movie represents 2.8 ns of simulation with a ligand pulling speed of 0.0025 Â/ps.

Video Protonation of H2S - Short.mp4

Movie S3. QM/MM modeling of deprotonation of H_2S in DosS heme pocket. Deprotonation of H_2S [shown in yellow (S) and white (H) spheres] following binding to the DosS Fe^{3+} heme iron is shown. The proton transfer to a nearby heme propionate group via a water bridge is spontaneous and occurs within 0.35 ps. The QM subsystem is depicted in balls and sticks. The overall DosS secondary structure is shown as a gray ribbon. Red = oxygen, Blue = nitrogen, Pink = heme center. Movie represents 0.4 ns of simulation.

SI References

1. D. W. Kraus, J. B. Wittenberg, J. F. Lu, J. Peisach, Hemoglobins of the Lucina pectinata/bacteria symbiosis. II. An electron paramagnetic resonance and optical spectral study of the ferric proteins. *J Biol Chem* **265**, 16054-16059 (1990).
2. V. Vitvitsky, P. K. Yadav, A. Kurthen, R. Banerjee, Sulfide oxidation by a noncanonical pathway in red blood cells generates thiosulfate and polysulfides. *The Journal of biological chemistry* **290**, 8310-8320 (2015).
3. A. C. Mot *et al.*, Fe(III) - Sulfide interaction in globins: Characterization and quest for a putative Fe(IV)-sulfide species. *J Inorg Biochem* **179**, 32-39 (2018).
4. D. W. Kraus, J. B. Wittenberg, J. F. Lu, J. Peisach, Hemoglobins of the Lucina pectinata/bacteria symbiosis. II. An electron paramagnetic resonance and optical spectral study of the ferric proteins. *J.Biol.Chem* **265**, 16054-16059 (1990).
5. T. Bostelaar *et al.*, Hydrogen Sulfide Oxidation by Myoglobin. *Journal of the American Chemical Society* **138**, 8476-8488 (2016).
6. Z. Palinkas *et al.*, Interactions of hydrogen sulfide with myeloperoxidase. *Br J Pharmacol* **172**, 1516-1532 (2015).
7. S. A. Bieza *et al.*, Reactivity of inorganic sulfide species toward a heme protein model. *Inorg Chem* **54**, 527-533 (2015).
8. F. P. Nicoletti *et al.*, Sulfide binding properties of truncated hemoglobins. *Biochemistry* **49**, 2269-2278 (2010).
9. H. Takahashi *et al.*, Hydrogen sulfide stimulates the catalytic activity of a heme-regulated phosphodiesterase from Escherichia coli (Ec DOS). *J Inorg Biochem* **109**, 66-71 (2012).
10. V. Fojtikova *et al.*, Effects of hydrogen sulfide on the heme coordination structure and catalytic activity of the globin-coupled oxygen sensor AfGcHK. *Biometals* **29**, 715-729 (2016).
